# Readsynth: short-read simulation for consideration of composition-biases in reduced metagenome sequencing approaches

**DOI:** 10.1186/s12859-024-05809-3

**Published:** 2024-05-15

**Authors:** Ryan Kuster, Margaret Staton

**Affiliations:** Department of Entomology and Plant Pathology, University of Tennessee, Knoxville, TN USA

**Keywords:** Metagenome, Simulation, RADseq, Reduced-representation, RMS, GBS

## Abstract

**Background:**

The application of reduced metagenomic sequencing approaches holds promise as a middle ground between targeted amplicon sequencing and whole metagenome sequencing approaches but has not been widely adopted as a technique. A major barrier to adoption is the lack of read simulation software built to handle characteristic features of these novel approaches. Reduced metagenomic sequencing (RMS) produces unique patterns of fragmentation per genome that are sensitive to restriction enzyme choice, and the non-uniform size selection of these fragments may introduce novel challenges to taxonomic assignment as well as relative abundance estimates.

**Results:**

Through the development and application of simulation software, readsynth, we compare simulated metagenomic sequencing libraries with existing RMS data to assess the influence of multiple library preparation and sequencing steps on downstream analytical results. Based on read depth per position, readsynth achieved 0.79 Pearson’s correlation and 0.94 Spearman’s correlation to these benchmarks. Application of a novel estimation approach, *fixed length taxonomic ratios*, improved quantification accuracy of simulated human gut microbial communities when compared to estimates of mean or median coverage.

**Conclusions:**

We investigate the possible strengths and weaknesses of applying the RMS technique to profiling microbial communities via simulations with readsynth. The choice of restriction enzymes and size selection steps in library prep are non-trivial decisions that bias downstream profiling and quantification. The simulations investigated in this study illustrate the possible limits of preparing metagenomic libraries with a reduced representation sequencing approach, but also allow for the development of strategies for producing and handling the sequence data produced by this promising application.

**Supplementary Information:**

The online version contains supplementary material available at 10.1186/s12859-024-05809-3.

## Background

Since its first application, reduced metagenomic sequencing (RMS) has remained a niche approach to profiling microbial communities. First coined by Liu et al., RMS is the application of reduced-representation sequencing (RRS) to metagenomic libraries, adopting steps modified from the original ddRADseq protocol [1, 2]. Metagenomic profiling of human samples comparing RMS and whole genome sequencing (WGS) yielded similar microbial profiles, and concerns of GC content bias weren’t detected. Three further studies have shown the potential for RMS to yield similar results or even outperform the traditional 16S and WGS approaches [3–5].

Although traditional approaches of amplicon and WGS can be effective for studying community structure, they exist on extreme ends of sequencing efforts and the benefits of each may exist in a middle ground. The conserved gene regions used in amplicon sequencing, often the 16S or ITS gene, can create bias towards some community members due to the primers selected and often these marker gene targets lack the resolution to consistently identify one species or strain from another [6]. WGS increases resolution but is comparatively expensive for deep sampling of rare taxa. Continuous, overlapping reads originating from closely related taxa may be computationally challenging to assign. Comparable to genotyping by sequencing (GBS), RMS reduces a genome into sampled DNA fragments by using one or more restriction enzymes [7]. These subsetted fragments represent predictable, targeted loci within a genome and function as hundreds of markers within each microbial genome allowing for species and even strain-level identification not found in amplicon sequencing alone. RRS increases per-locus sequencing depth for these fragments and allows for more samples to be analyzed in the same sequencing run, improving sequencing accuracy and lowering the per-sample cost of sequencing [1, 8, 9]. Unlike the original application of reduced representation sequencing for GBS, which produces fragments from the genome of an individual, RMS creates fragments from many unknown source genomes into a single pooled sequencing run.

Despite the potential benefits of RMS, the ability of this approach to accurately quantify at the species and strain level has not been tested at scale, possibly hindering adoption by metagenomics researchers. A major caveat to the RMS approach is that restriction enzyme motifs are enriched in a taxa-dependent manner [3]. Genome size, which can range over an order of magnitude in bacteria, as well as the varying degrees of sequence conservation among closely related microbes may complicate RMS quantification [10]. For example, genomic loci yielding RMS fragments may be variable even among closely related individuals, as a single base mutation altering a restriction cut site can lead to “allelic dropout” [11]. The degree to which this variation affects taxonomic identification and determination of relative abundance of community members has not been studied deeply. Every community member comprising the metagenomic sample will yield a unique distribution of fragment lengths.

Further complicating RMS profiling, sequencing effort (i.e. total read count) and size selection constraints may significantly impact the taxa included in the final sequencing library. Both PCR amplification and fragment size selection steps will affect the probability of a given DNA fragment surviving into the final sequencing reaction in a size-dependent manner with possible PCR biases dependent on template length [12, 13]. Gel or bead mediated size selection is a critical step in RRS library design that helps to remove adapter dimers and optimize flow cell performance, and so remains an unavoidable library preparation step in most cases. An alternative RRS approach using *isolength* (type IIB) restriction enzymes has been proposed as a potential solution to this issue of variable fragment length [14, 15]. Using a single enzyme that produces constant fragment lengths frequently across the genome may create detailed fingerprints of genomic communities without size selection steps, assuming adapter dimers can be reduced. Normalizing for the non-uniform read depths associated with a single organism is a task that existing metagenome profiling software aren’t designed to handle.

In applications such as RMS, where existing short read approaches are being used in novel ways, it may be difficult to gain traction because the features of the data are so understudied for the intended application. Spending money to develop new experimental techniques can be a highly risky endeavor, and it also requires the development of custom software tools and statistical approaches necessary to analyze this new form of data. In instances such as these, simulation can be useful to predict and overcome artifacts from library prep that could not be anticipated or otherwise measured without a ground truth. Capturing the nuances of RMS behavior under different conditions is a necessary step in understanding its application, therefore, we introduce the software *readsynth* as a simulation aid to researchers in considering these promising alternative approaches to sequencing metagenomes. Readsynth simulates read count constrained metagenomic sequence data based on pre-assembled genomes and user-defined community compositions along with multiple library preparation parameters.

In this study, both real and simulated RMS libraries are investigated to understand the influence of non-uniform fragment sizes and possible molecular and computational considerations that might be used to overcome these biases. Bias in fragment lengths may be occurring due to the biology of the individual community members being sampled and/or the technical library preparation. These factors prevent the total numbers of reads mapped to reference genomes from being directly used for calculating relative abundance. Our proposed solution uses the *fixed length taxonomic ratio* (FLTR) of read depths occurring within a non-uniform fragment length distribution. Barring the presence of strong GC bias, the ratio of two taxa should remain constant within all fragment lengths where both taxa are present. We first benchmark readsynth’s ability to faithfully capture features of mock community data. To demonstrate the utility of readsynth simulations, we then investigate this novel FLTR quantification approach that may overcome some of the challenges produced by non-uniform fragment distributions. Upon finding the library conditions that produce the best resolution, we consider the ability of these approaches when the search space includes a full database of all available reference genomes.

## Implementation

### Simulation software overview

**Fig. 1:**
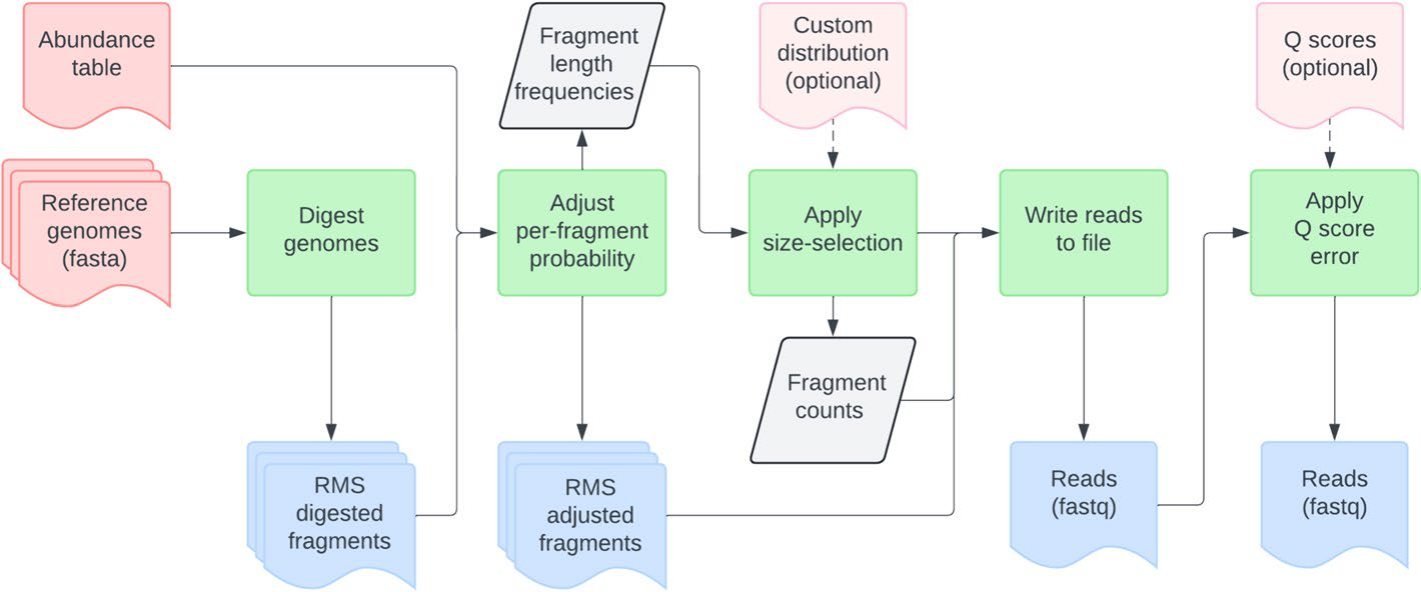
Overview of readsynth inputs and flow of data. Necessary input files (shown in pink) are a collection of genome files and a corresponding abundance table for each. Output files include per fragment count estimates and the final, paired-end read fastq files

Readsynth was developed to simulate Illumina short read libraries to assess the compositional abundance of highly custom communities under multiple reduced sequencing conditions. Readsynth is a command line software package written in Python and C++ that uses commonly maintained statistical packages and consists of a digestion, size-selection, and read-writing stages (Fig. 1). The software was written to be highly customizable across three categories: (1) microbial community composition, defined by input genomes and their relative abundance; (2) experimental parameters, including reduced library approach, enzyme digestion rates, expected fragment length distributions, and custom adapter design, and (3) sequencing parameters, such as total read number, read length, and base quality value profiles.

#### Digestion

Readsynth first reads each input genome assembly individually to capture the set of possible fragments and calculate the probability of each sequence fragment surviving to the final library. Given a user input set of IUPAC restriction site motifs, overlap-tolerant regular expression (regex) searches are performed to exhaustively detect all possible cleavage sites and define fragments within the expected size-selection distribution. Fragments resulting from any combination of palindromic restriction enzyme motifs are modeled probabilistically to account for partial enzyme digestion. The probability of a fragment remaining at the end of digestion is calculated based on the probability of an enzyme cut producing the necessary forward and reverse adapter-boundary sites, adjusted accordingly for fragments harboring internal cut sites.

$$Pr\left( {fragment} \right) = \left\{ {\begin{array}{*{20}l} 1 \hfill & {if c = 1 and i = 0} \hfill \\ {c^{2} \left( {1 - c} \right)^{i} } \hfill & {otherwise} \hfill \\ \end{array} } \right.$$

The per-fragment probability is a function of enzyme cleavage occurring at both ends of a sequence based on a user defined enzyme cut efficiency (c). Sequences that harbor greater numbers of internal cut sites (i) are less frequently represented as a sequenced read. The probabilities for each fragment length are then summed, approximating the expected fragments given a single genome copy for each genome in consideration.

#### Size selection

**Fig. 2:**
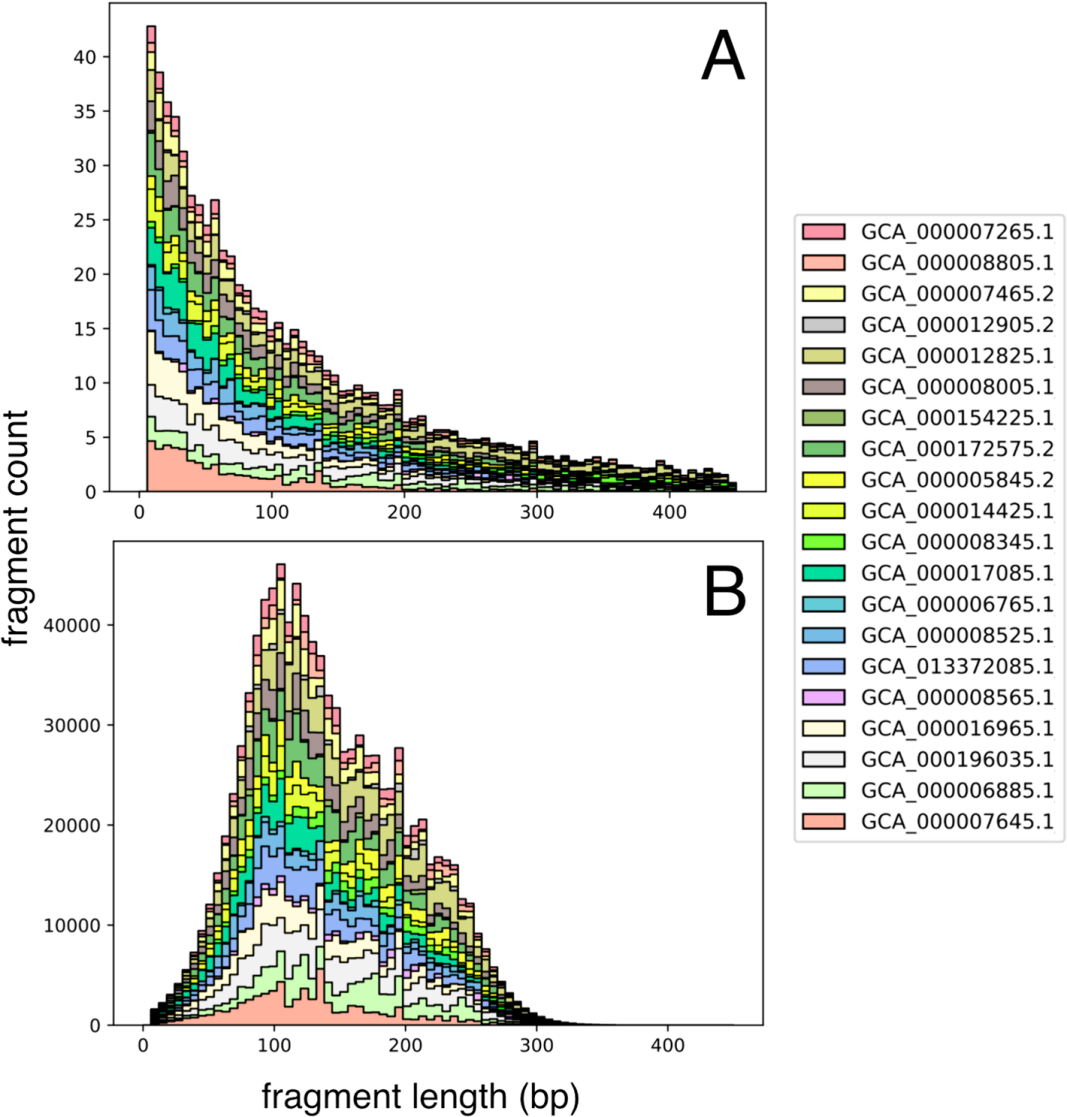
Digestion distribution of reduced metagenome sequencing (RMS) fragment lengths (bp) **A** after simulated enzyme fragmentation, counts represent expected fragment frequency with 1X genome coverage; **B** final read counts of size-selected fragments from the intersection of a Gaussian normal (μ: 150 bp, σ: 50) and A, scaled to the count at length 100 bp from A. Bar colors indicate individual bacterial genomes present in the simulated community

To simulate size selection, each fragment’s post-digestion probability is adjusted based on multiple additional factors affecting its representation. First, the expected fragment counts for each genome are scaled by their proportional abundance, as defined in the abundance table. The combined distribution of digested fragment lengths for all input genomes then undergoes size selection. The counts of the digestion distribution are used to scale a Gaussian probability density function at a given length, x, and this intersection of sample spaces defines the final size selection distribution (Fig. 2). This approach follows the size variability expected in gel-based size-selection equipment (e.g., SageScience BluePippin) at the narrow and broad range selection techniques while preventing artificial inflation of reads in lower abundance than produced by the Gaussian curve [1]. To simulate the hardware-imposed limitation on the composition of metagenomic fragments, the input read number (n) is divided evenly amongst the resulting size-selected distribution of the digested metagenome.

#### Error modeling

Readsynth applies a straightforward substitution error model to every read using randomly sampled Q scores from any existing fastq file, with several publicly available profiles to select from. Phred-like error probability rates from the sampled Q scores are used to mutate each nucleotide base to a non-self modification using pseudo-random number generation. Simulated fragments that are shorter than the simulated read length resulted in expected adapter contamination in data output (Supplementary Fig. 1), and users may provide any number of custom-designed adapters with specific overhangs.

### Software benchmarking

In order to benchmark the simulation accuracy of readsynth, we use existing RMS data as a ground truth. The loci-specific enrichment of reads as well as the similarity in taxonomic profiles between real and simulated reads are used as performance metrics. Two previously sequenced mock community data sets produced with RMS approaches were considered. These communities used standardized concentrations of each community member, making them ideal for comparison with simulated reads. The first dataset from Snipen et al. consists of a Human Microbiome Project mock community of 20 bacterial strains (BEI HM-782D) digested using the restriction enzymes EcoRI and MseI [5]. To simulate the abundance of each taxon, the input abundance table used the reciprocal of the ribosomal copy number determined from the ribosomal RNA operon copy number database [16]. The second dataset from Sun et al. used a separate community of 20 bacterial strains (ATCC MSA-1002), which was assumed to have even genomic copy number [14]. This dataset was created using the type IIB restriction enzyme BcgI which produces short fragments with no length variation.

#### Extracting sequencing features from real and simulated mock communities

Sequence reads from the existing mock communities were adapter trimmed using Cutadapt 4.1 [17]. Reads were then mapped against each of the community member reference genomes using BWA MEM (Burrows Wheeler Aligner 0.7.17) [18]. Samtools 1.15.1 was used to select only paired end reads with the appropriate orientation. The Samtools stats and depth commands were used to summarize the read lengths as well as the per-position depth of all aligned reads [19]. Using positional information from these reads, a custom fragment length distribution was estimated for use in simulation. The count ratio of fully digested fragments to the larger fragments immediately encompassing them (r) was used to estimate the cut efficiency (c) of enzyme digestion except for the case of complete digestion in which c = 1. In cases of incomplete digestion, following the per-fragment probability model, fragments containing internal cut sites will occur less frequently than their contained fragments such that r must be greater than 1.

$$c = \left\{ {\begin{array}{*{20}l} {\frac{{\left( {r - 1} \right)}}{r}} \hfill & {1 < r < \infty } \hfill \\ 1 \hfill & {complete digestion} \hfill \\ \end{array} } \right.$$

Only fragment lengths in the range of 100 bp to 450 bp were used to estimate cut efficiency, as these were observed to be less constrained by size selection and therefore more reliable in preserving true read ratios (Supplementary Fig. 2). Aligning community mixtures of many taxa against individual reference genomes in BWA MEM returned many reads that aligned to multiple genomes (Supplementary Fig. 3). Further, variability between the published reference sequence and the real sequence data resulted in the rare presence of RMS fragments not reproducible in simulation. In order to make meaningful comparisons between the real and simulated data, custom Python scripts were used to extract positional information from high quality read alignments in the sam-formatted file in order to preserve the fragment size distribution while removing duplicate alignments.

Simulation was performed using fragment size distribution, enzyme cut efficiency, and Q score profile derived from the real sequence data. Although we expect these derived inputs to most closely resemble the real data, additional simulations were run to test the importance of fragment size distribution and enzyme cut efficiency. Three fragment length distributions were considered to measure the impact of size selection. The first was created using the exact fragment counts extracted as a custom.json file from the extracted sequence alignments. The second simulated size selection using the mean and standard deviation from the samtools summary statistics to define the distribution shape. The third size selection distribution used identical standard deviation values, but with the mean fragment size increased by 100 bp. BWA MEM and Samtools were again used to map simulated reads to the reference genomes and the read depth at every expected fragment position was counted. Cumulative read depth across every position in the mock community was compared between real and simulated sequences using both Pearson and Spearman correlation.

#### Assessing compositional-biases using simulation

Simulations of RMS sequencing reads were informed using the mock community and two representative gut metagenomic communities based on sequencing efforts from complex microbial samples. Mock community sequences from Snipen et al. (replicates SRR10199716, SRR10199724, and SRR10199725) were again used as a baseline to quantify taxonomic abundances from a mixture of 20 bacterial taxa at known relative abundance. Taxa were assumed to be approximately equimolar in ribosomal operon count as described in the product specifications. A single human stool sample (SRR5298272) prepared with RMS using NlaIII and HpyCH4IV was used to establish a microbial community with greater richness for simulation [2]. These biological sample reads were assigned putative taxonomic labels using Kraken2 and Bracken (Standard plus protozoa, fungi & plant database ‘PlusPFP’ June 2022) and resulting taxonomic identification numbers were used to download representative genomes from each of the 691 non-host hits [20, 21]. Finally, the OTU profile resulting from gut samples from 2,084 individuals from the Healthy Life in an Urban Setting (HELIUS) study served as a basis for simulating microbial communities with authentic taxonomic abundances [22]. Of the 744 OTUs, 610 unique RefSeq genome references from the genus and species level served as the basis for simulation. When multiple OTUs from the same genus level were encountered, multiple species from this genus were selected based on genomes with full, major releases in GenBank, which included many highly similar strains to be simulated in the same community. All simulations were performed using the software readsynth (0.1.0; commit 88d8bb1).

To assess the sensitivity of RMS to capture rare taxa, metagenomes based on HELIUS communities were simulated using a series of increasing total read counts (1 × 10^5^, 1 × 10^6^, 1 × 10^7^, and 1 × 10^8^ paired end reads) and four combinations of restriction enzyme double digests. The combinations of restriction enzymes selected (EcoRI/AgeI, EcoRI/MseI, HhaI/AgeI, and HhaI/MseI) were chosen for the diversity of the cut site frequency and GC content of the recognition motifs (Table 1). Additionally, a type IIB enzyme “BcgI” digest was simulated to assess the application of the resulting isolength fragments.

**Table 1:** Combinations and expected characteristics of the restriction enzymes selected for double digests in HELIUS-inspired RMS simulations

Forward	Reverse	Frequency	GC content
EcoRI (G/AATTC)	AgeI (A/CCGGT)	Rare—rare	Even
EcoRI (G/AATTC)	MseI (T/TAA)	Rare—common	GC poor
HhaI (GCG/C)	AgeI (A/CCGGT)	Common—rare	GC rich
HhaI (GCG/C)	MseI (T/TAA)	Common—common	Even

Paired end reads from each of the real and simulated datasets were aligned to a combined reference genome concatenated from all known members in the metagenome using BWA MEM. Mapped reads with a MAPQ score of zero were removed to avoid reads that map closely to multiple reference genomes. Custom Python scripts (available at github.com/ryandkuster/readsynth_analysis) were used to recreate the original genomic fragments corresponding with each read pair based on the simulated start and end positions for each fragment produced in simulation. Only those fragments harboring no internal cut sites were kept as incomplete digests are expected to unpredictably affect quantification and assessment of existing RMS data found these fragments to be rare. The observed count and corresponding taxonomic assignment were stored for each fragment.

We also assess the performance of RMS data when the reference database is not curated to only those references in the ground truth set, as is the case in most practical applications of metagenomic profiling. Reads simulated using taxa derived from the Liu et al. RMS stool sample dataset were queried against the Kraken2 ‘PlusPFP’ database. We wanted to see if mapping and recreating fragments using an inclusive database would produce taxa count and abundance comparable with the input 691 taxa simulated at even relative abundance.

#### Fixed length taxonomic ratios

**Fig. 3:**
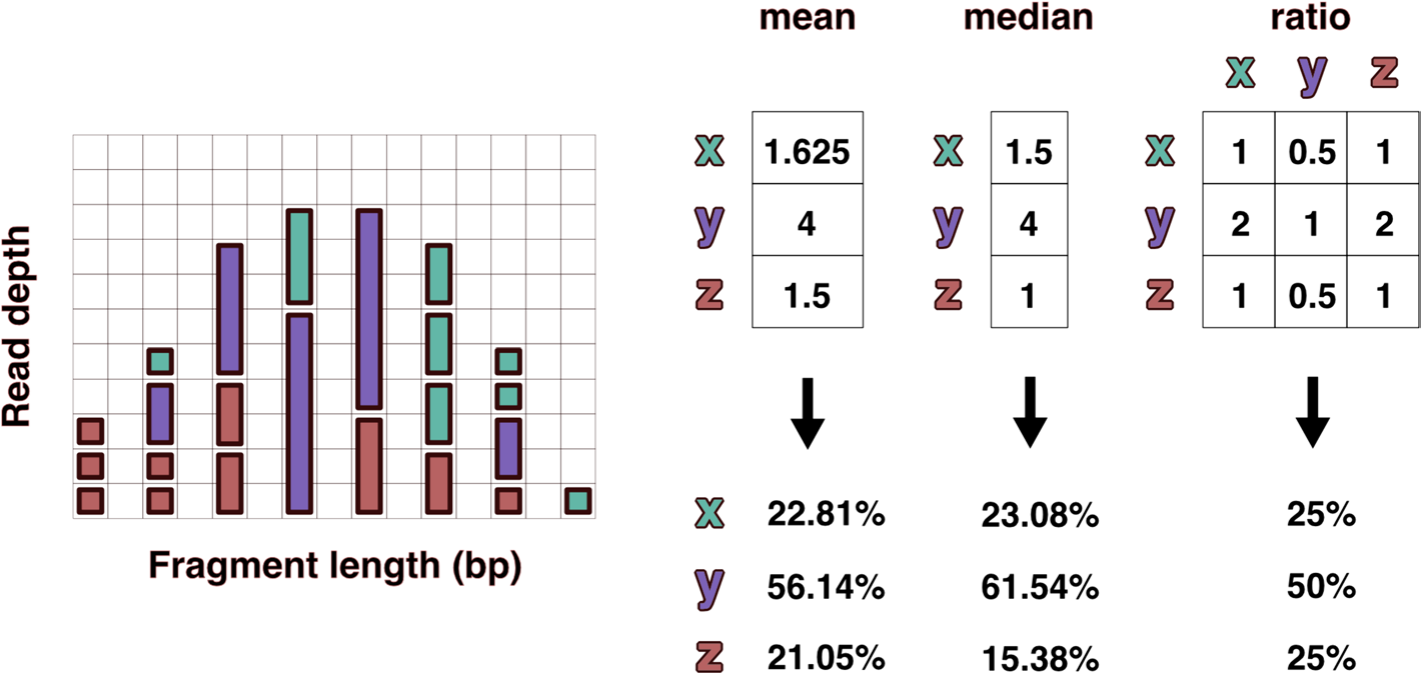
Visual representation of read depths originating from hypothetical taxa x, y, and z present in a 1:2:1 ratio. The left distribution shows fragments resulting from reduced sequencing under size selection or PCR fragment length biases. The differences in relative abundance estimates produced using mean and median read depths is compared with the taxonomic ratio approach (FLTR) introduced here. The ratio table on the right displays the averaged pairwise read depth ratios calculated individually within each fragment length. Even assuming no bias within each fragment length, the mean and median read depth estimates produce relative abundances that don’t account for variance in the size distribution, which is often not normal or uniform

The frequency of fragments was analyzed individually across the range of observed fragment lengths. Within each discrete fragment length, the ratios of the observed counts between all taxa present were calculated. The ratio was calculated iteratively by dividing the individual fragment count by the average fragment count for each of the other taxa present at that length (Fig. 3). These taxonomic ratios were then averaged over all fragment lengths to produce an *n* x *n* matrix of all n taxa. Because many taxonomic ratios were missing, all columns in the taxonomic ratio matrix were scaled to the taxon with the greatest number of relationships to all other taxa. The row average of this scaled matrix was used to predict relative taxonomic abundance for all taxa.

## Results

### Performance of software

A hypothetical gut microbiome was established to assess the computational performance of readsynth on larger metagenomic communities. Reference genomes derived from human gut samples (Liu et al. [2]) were downloaded and simulated under various input settings using a single core on a AMD EPYC 7F72 24-Core Processor (x86_64, 3028.149 MHz) (Supplementary Table 1). The cumulative size of the metagenome reference sequences influenced the time to simulate, but choice of enzymes had the largest impact on time and performance. Frequent, 4-base cutters produced many potential fragments to process, and use of less frequent motifs performed more efficiently. The most time consuming task tested, two 4-base cutters against 691 microbial genomes and the human genome, took 204 min; all other tasks took less than two hours.

### Benchmarking of simulation accuracy

**Fig. 4:**
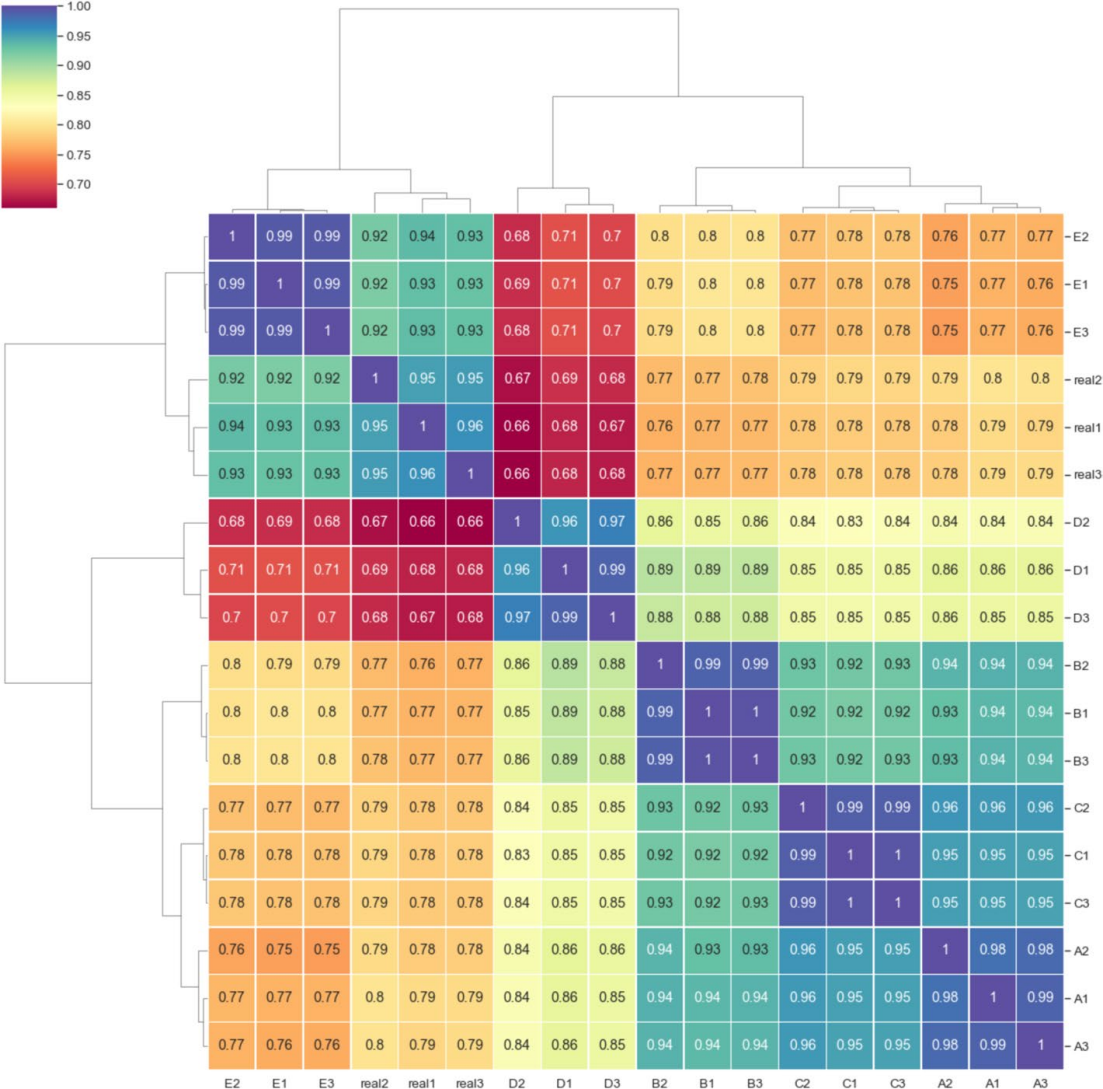
Spearman correlation coefficient of metagenome-wide, position-specific read depth for each of three replicates of real and simulated mock community sequencing. Real1-3 are the depth correlations of the real sequencing data replicates. Mapped read depth correlations from readsynth simulations are shown in **A**–**E**: **A** custom (.json) dictionary of fragment lengths derived from real sequence data and cut efficiency derived from real sequence data;** B** normal distribution of fragment lengths and cut efficiency derived from real sequence data; **C** normal distribution of fragment lengths with fragment mean increased by 100 bp and cut efficiency derived from real sequence data; **D** normal distribution of fragment lengths and cut efficiency reduced to 0.8; **E** normal distribution of fragment lengths and cut efficiency increased to 1

Spearman correlation captured a monotonic relationship between real and simulated read depths across the length of all 20 reference genomes in the mock community, and therefore was sensitive to differences in fragment presence or absence. The reads simulated using 100 percent enzyme cut efficiency corresponded closely with the real data (Fig. 4 group E; r = 0.92 to 0.94), comparable to the covariance measured between replicates of the real data. Simulation with a lower enzyme cut efficiency of 80 percent produced lower correlation with the real data, possibly resulting from increased novel loci surviving the fragmentation process (Fig. 4 group D, r = 0.66 to 0.68).

Pearson correlation was sensitive for comparing fragment read depths and was affected by noise in the data, as can be seen by variation within the replicates of the real sequencing datasets. The simulation that produced the highest read depth correlation coefficients between 0.76 to 0.79 used exact fragment size distributions informed by the real dataset (Supplementary Fig. 4, group A). Using a normal distribution produced coefficients between 0.71 and 0.76 (groups B, D, E), suggesting the real data was approximated by applying readsynth’s normal distribution approach. The distribution with mean fragment lengths shifted only 100 bp longer affected read depth considerably with coefficients of 0.67 to 0.7 across replications (Supplementary Fig. 4, group C). Simulations of the isolength dataset produced lower Pearson correlation (r = 0.56) but higher Spearman correlation (r = 0.97). The use of type IIB enzymes avoids variability in fragment length and while simulations with readsynth aligned closely with sequence presence-absence, simulation did not capture variability in the per-fragment depth.

### Comparing Kraken2/Bracken profiles of real and simulated mock communities

**Fig. 5:**
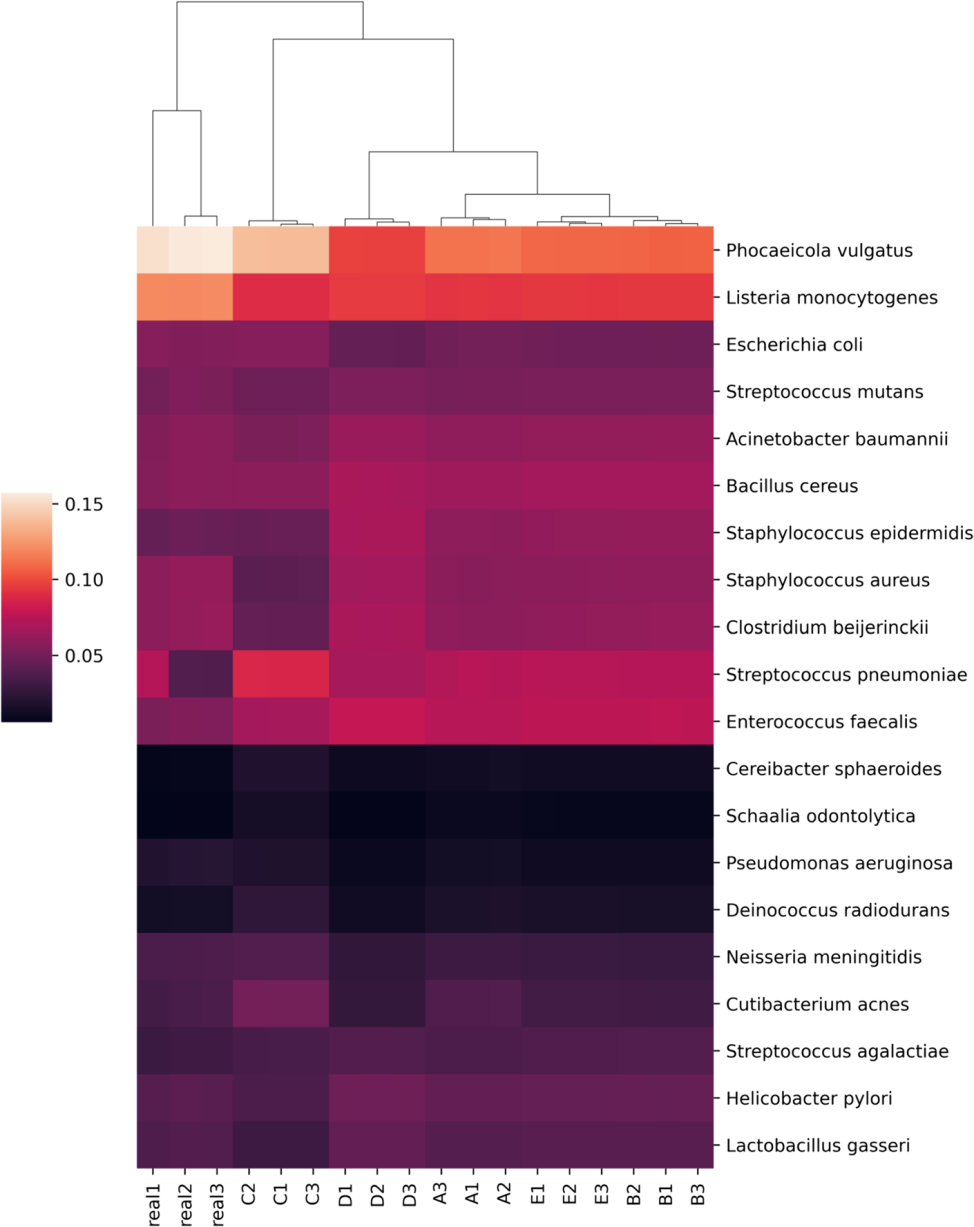
Kraken2/Bracken percent abundance profiles based on read assignments of real and simulated mock community sequencing. Real1-3 are the relative abundances of mapped reads per species produced from the real sequencing data. Mapped read depth correlations from readsynth simulations are shown in **A**–**E**: **A** custom (.json) dictionary of fragment lengths; **B** normal distribution and cut rate; **C** normal distribution and cut rate with fragment mean increased by 100 bp; **D** normal distribution and cut rate with cut rate reduced to 0.8; **E** normal distribution and cut rate with cut rate increased to 1. All fragment distributions and cut rates estimated from real sequence data unless specified

The adapter-trimmed reads from the previously described mock community sequencing efforts were profiled using custom databases with Kraken2 (2.1.2) and Bracken (2.7). Raw reads mapping to each species-level identification was used as a metric of performance between the real and simulated reads. Generally, simulations captured trends in the distribution of real sequence data accurately (Fig. 5). The simulations with increased fragment size selection and decreased cut efficiency (Fig. 5, groups C and D) caused larger shifts in predicted distributions. These changes are largely due to taxa-specific patterns of fragmentation where some community members, such as *Phocaeicola vulgatus* and *Staphylococcus epidermidis*, contain disproportionate read counts originating from either long or short fragments (Supplementary Fig. 5).

### Impact of simulation parameters and quantification method

The FLTR approach is an alternative to read median or mean depth for profiling that measures the average relationships in read depth ratios between the taxa present at a given length. To test the FLTR approach, we first compared it to mean depth and median depth using the Snipen et al. 2021 mock community samples. Across the three replicates, using ratios produced relative abundance estimates similar to those produced using either the median or mean read depths, with Pearson correlation to the ground truth relative abundances ranging between 0.809 and 0.852 (Supplementary Fig. 6). Estimates of relative abundance across the three replicates show consistent, taxa-specific patterns of over- and underabundance in several of the 20 mock community members (Supplementary Fig. 7). These differences may be the result of sequence-specific amplification biases or slight deviations from the reported proportions expected from the mock community standard.

**Fig. 6:**
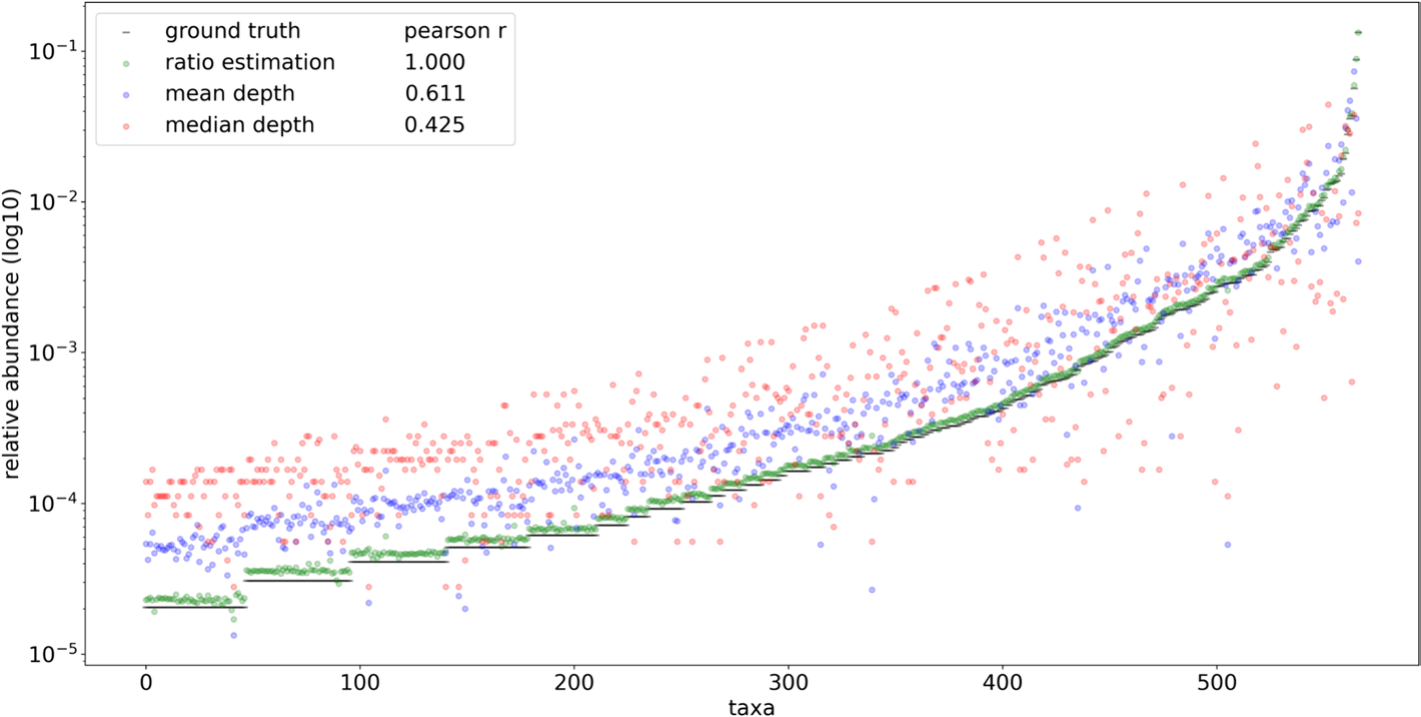
Comparisons of ground truth relative abundance (black) vs. results obtained using taxonomic ratio approach (green), mean depth (blue), and median depth (red) for 567 taxa returned using HELIUS simulated reads digested with HhaI and AgeI using 1 × 10^8^ total reads

**Fig. 7:**
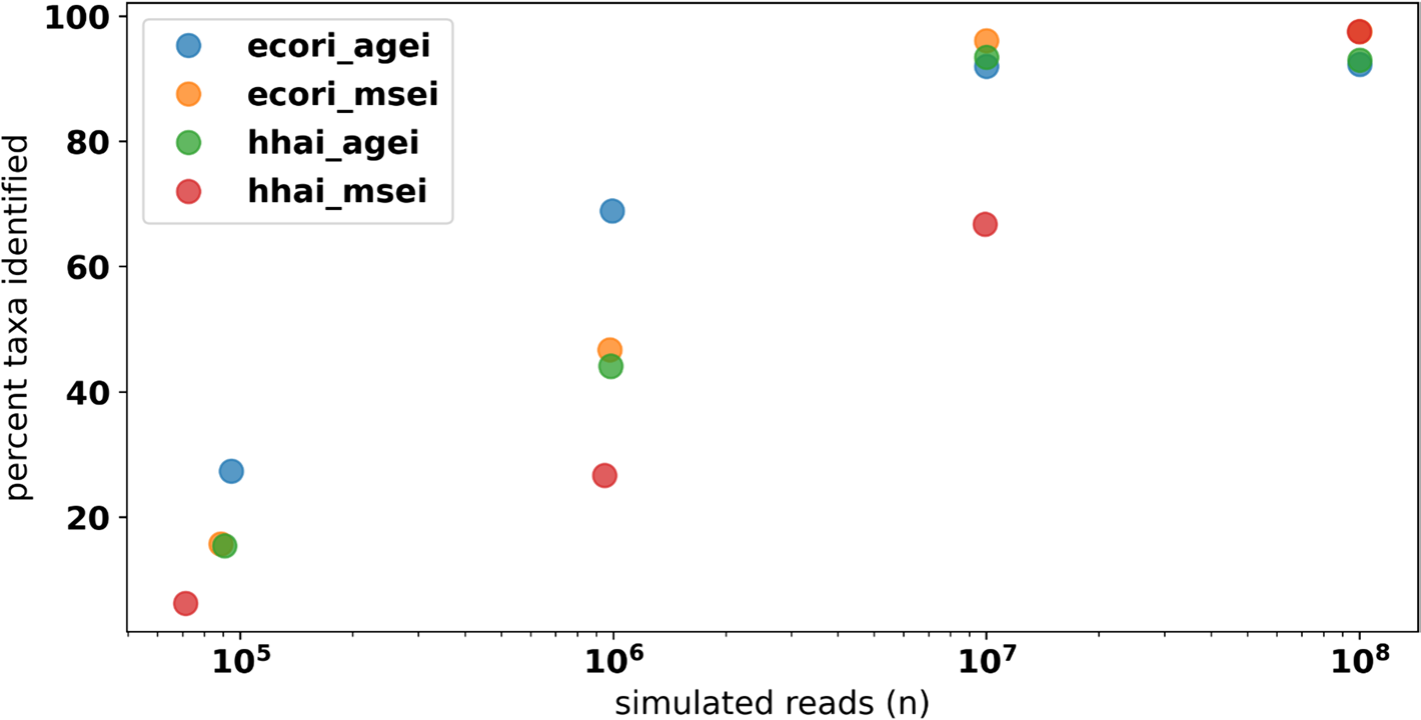
The relationship between the input simulated reads target and the percent of taxa identified across 4 combinations of restriction enzyme double digests. Of the expected 610 unique taxa, many are not captured at lower levels of coverage. Combination of rare cutters (EcoRI and AgeI) performed better at these lower levels of coverage

Data simulated from the 610 HELIUS gut microbial taxa were used to assess differences in profiling performance for the new FLTR approach versus previously published methods under varying library preparation conditions. Across all simulated HELIUS datasets, FLTR estimates of relative abundance outperformed the mean and median read depth in every instance (Supplementary Table 2). In some extreme cases, relying on the mean and median depths fall far from the target relative abundances (Fig. 6). Reads simulated from the HELIUS data captured several general trends not captured with the mock community analyses. Most notably, we see a strong interaction between the characteristics of restriction enzymes selected and the signal of the taxa simulated. Use of two frequent restriction enzymes, HhaI and MseI, each with 4 bp recognition motifs, required 100 million reads in order to detect > 90% of the input taxa. Interestingly, the use of two infrequent restriction enzymes, EcoRI and AgeI, was able to identify a higher percentage of taxa at lower sequencing efforts relative to the other treatments considered (Fig. 7). At 10 million reads, the combination of EcoRI and MseI captured 96.1% of taxa. The majority of simulated datasets performed moderately well at 10 million reads, and this was used as the basis for additional simulations. Lowering the mean fragment size for the EcoRI/MseI simulations by 100 bp reduced the number of identifiable taxa to 89%, and an increase by 100 bp marginally improved detection to 96.7%. Simulating the isolength, BcgI fragmentation of the HELIUS data returned only 56.1% of the 610 taxa, compared with the 66.7% of taxa identified by HhaI/MseI at the same sequencing effort (Supplementary Fig. 8). Across all simulations at 10 million reads, between 16 and 20% of reads were discarded per simulation due to the multi-mapping criterion described in the methods.

**Fig. 8:**
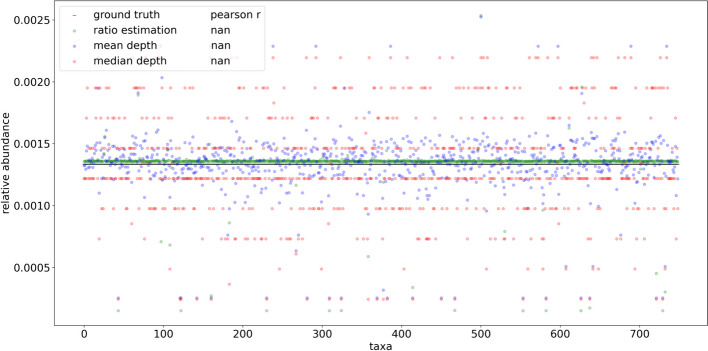
Comparisons of ground truth vs. results obtained using taxonomic ratio approach, mean depth, and median depths by mapping simulated reads naively to a fully inclusive, pre-made Kraken2 database (‘PlusPFP’) before fragment recreation. A total of 749 taxa were returned using this approach. Reads from 691 taxa were simulated based on the metagenomic taxa from Liu et al. [2] sequence SRR5298272 using even representation and 1/749 was used as ground truth to reflect the assumption of equal abundance. In silico reference genomes were digested with NlaIII and HpyCH4IV producing ~ 3.3 × 10^6^ total reads

In order to compare the profiling performance of RMS reads when mapping to a known set of reference genomes and a fully inclusive database, 691 taxa based on the Liu et al. dataset were simulated using even abundance. Using even relative abundance allows qualitative assessment of profiling quantification efforts independent from taxonomic assignment, which expectedly contain naming and identification discrepancies between the input reference genome naming and the resulting assignments. Unlike the HELIUS results, aligning these reads to the known set of references was able to uniquely identify every input genome (Supplementary Fig. 9). The same simulated reads were then assigned to the species level of the ‘PlusPFP’ Kraken2 database using Bracken, which includes the target genomes and an additional 28,763 non-target genomes at the species level or lower. The resulting number of taxonomic hits was inflated from 691 to 1534 total taxa. To explore the basis of these false positive taxonomic assignments and potential methods of reduction, the reference genomes from these preliminary hits were then used for BWA MEM alignment and fragment recreation in place of the curated, known reference approach described above. Upon selecting reads whose source fragments aligned to the expected cut positions and removing multi mapped reads, 93.8% were able to map precisely to fragments expected in 749 of the 1,534 taxa from the Kraken2/Bracken profile, greatly reducing the false positives. Visual inspection of the recreated fragment length distribution captured the expected profile (Supplementary Fig. 10). Applying the FLTR approach to these reads yielded broadly even estimates of relative abundance matching the expected community composition. Some of the estimates have much lower than even representation, but comparatively these results further support the fallibility of using mean and median read depths to estimate abundances using RMS (Fig. 8).

## Discussion

Simulation is a meaningful way to measure the behavior of bioinformatics approaches, but its utility hinges on its ability to faithfully capture features found in real sequencing data. The applications of short reads are highly variable. Often the impacts of library prep are overlooked in the production of bioinformatics tools, and more tools need to be considerate of these possible nuances. Readsynth simulations of RMS mock communities produced realistic taxonomic distributions of genomic fragments and the output sequence profiles corresponded closely to those found in the limited RMS mock community sequences that exist. Each taxon present in the simulated RMS mock community produces a unique digest profile at varying relative abundance, and readsynth simulations faithfully captured these influential patterns in read depth across all combined loci in the metagenome. These simulations also demonstrate the influence that library preparation can have on profiling efforts. The fragment origin and read depth were sensitive to even minor differences in simulated library parameters, particularly size selection and enzyme efficiency.

Restriction enzyme digests of metagenomic communities produce irregular fragment length distributions that cannot be readily modeled assuming features of an underlying distribution. Therefore, calculating relative proportions in the context of a mixture of individuals prepared this way is a challenge that has not been properly addressed to account for the influences of library preparation. The FLTR methodology proposed here may benefit efforts in metagenomic quantification when fragment or target length differs between organisms. It may also have useful applications outside of metagenomics where fragment size bias affects abundance estimates using read depth, such as genotyping approaches using reduced representation sequencing. We found that using the FLTR approach appears to be a more stable metric than either mean or median depth, often because RMS fragments are not unimodally distributed within an individual genome. Removal of multi-mapped reads may compound this effect by leaving only a small number of reads representing a genome, and these reads may exist in regions of the fragment length distribution that are influenced by size-selection and PCR length biases. For RMS approaches to overcome the confounding influence of variable fragment lengths, it is a necessary prerequisite to first recreate fragments in order to know fragment lengths. Fortunately, if a reference genome assembly is reliable enough to produce a strong hit to RMS reads, it should be able to provide a framework for simulating the expected fragments so long as the cut sites are preserved. Conversely, reads that remain unassigned or ambiguous cannot be interpreted as proportional due to the uneven contribution of community members, the removal of which may result in inflated estimates of relative abundance. It may be possible to retain multi-mapped reads using estimated abundances of uniquely aligned kmers, such as the KrakenUniq approach to assignment. Such approaches have not been tested in this study.

Simulated assessments of RMS’s capability to capture rare taxa suggest that it may be possible but is highly dependent on the library preparation methods. Given a finite set of sequence reads, the restriction enzymes selected and size sampling protocols will determine which taxa produce enough signal to be detected. Fragmenting a set of highly diverse genomes with a frequent cutting enzyme may produce hundreds of thousands or even millions of potential fragments, but when these reads are distributed between taxa whose relative abundances differ by orders of magnitude, the signal for rare taxa may be lost. It is likely that enzyme digests that produce shorter fragments will create more multi-mapping collisions, as the shorter read lengths reduce mapping confidence. Here we found that digests using HhaI/MseI or the BcgI isolength enzyme produced the greatest number of potential fragments, but based on the sensitivity of the profiling approach employed, few were actually informative because the short fragments they produce often lack resolution against a highly inclusive database. This mapping-based phenomenon may also explain the success of infrequent cutters, which captured a greater percent of the taxa present using a fraction of the overall fragments as the frequent cutters. Increasing sequencing effort may allow for greater resolution when many fragments are produced; however, using upwards of 100 million reads per sample to capture rare taxa may reduce the cost benefits of RRS. Therefore, it is recommended to consider each community’s complexity when considering the necessary read coverage, as has been similarly proposed for WGS profiling [23].

When two closely related taxa exist within a community, simulations indicate that it may become difficult to estimate the relative abundance of each using the RMS approach. This is because most fragments originating from both organisms will be identical and only a small fraction of loci will be uniquely informative to a taxon. In instances when only a small number of fragments may be used for quantification, abundance estimates using read depths may become highly sensitive to per-fragment biases originating from size-selection or PCR. The development of ddRADseq protocols for GBS were based around comparing conserved regions of the genome between closely related species. With RMS, conservation between these fragments may be counterproductive when trying to parse the individual members of a complex community. The taxonomic ratio methodology described here does not rely on normalization, but it is also not designed to handle redundancies in multi-mapping fragments, as evidenced by the set of unidentified taxa in the simulated HELIUS community. Eliminating fragments based on similarity may ultimately eliminate all useful, identifying markers if multiple, nearly identical strains are present. Retaining these fragments requires a means of identifying distinct taxa when aligning against a fully inclusive database of potential matches, and the results may be highly dependent on the database as well as the behavior of the aligning tool used [24]. We also recommend future investigations into profiling software choice and handling ambiguously assigned reads.

While simulation cannot be expected to capture all the nuances of real sequencing data, it can help find the edge cases where existing tools might fail to perform as intended. Using a set of largely pre-existing bioinformatics tools, our assessments here of simulated RMS data may be successful in some instances and very underpowered in others. While RMS offers promising applications, profiling benchmarks have not been widely tested on mixed samples including viral, protist, or non-fungal eukaryotic members and instead focus largely on prokaryotic and fungal taxa. Simulation could be a useful means in determining whether such mixed communities contain enzyme site biases preventing meaningful profiling accuracy. RMS may provide a fast and affordable profiling technique for communities that are relatively simple in structure. It may also be fast and economical in instances where detecting rare taxa is not critical. One of the largest obstacles preventing community use of RMS is the lack of bioinformatic tools developed to handle the data it produces, and applying existing profiling tools will not work out of the box. Both developers and users of new tools should be cognizant of the intersection between sample preparation and downstream analytical tools selected.

### Availability and requirements

Project name: Readsynth

Project home page: github.com/ryandkuster/readsynth

Operating system(s): Linux/MacOS, 

Programming language: Python3, C++ 

Other requirements: Python packages: numpy, pandas, and seaborn

License: Apache-2.0

Any restrictions to use by non-academics: none.

### Supplementary Information

Additional file1 (DOCX 21675 KB)

## Data Availability

The code and parameters used in all simulation and analytical steps used in this study are available on GitHub at https://github.com/ryandkuster/readsynth_analysis and raw data are stored at https://doi.org/10.5061/dryad.nzs7h44zk. The freely available package readsynth can be downloaded at https://github.com/ryandkuster/readsynth. The mock RMS and isolength sequencing datasets analyzed for this study can be found in the NCBI BioProject PRJNA574678 https://www.ncbi.nlm.nih.gov/bioproject/PRJNA574678 and figshare https://doi.org/10.6084/m9.figshare.12272360.v8. The RMS gut metagenome data is available in NCBI BioProject PRJNA377403 https://www.ncbi.nlm.nih.gov/bioproject/PRJNA377403. The Kraken2/Bracken PlusPFP database (6/7/2022) and indices used in this study were downloaded from https://benlangmead.github.io/aws-indexes/k2.

## Abbreviations

RMS: Reduced metagenomic sequencing
RRS: Reduced representation sequencing
ddRADseq: Double-digest restriction-site associated DNA sequencing
GBS: Genotyping by sequencing
PCR: Polymerase chain reaction
OTU: Operational taxonomic unit
