# Supplementary material for: Readsynth: short-read simulation for consideration of composition-biases in reduced metagenome sequencing approaches
Source: BMC Bioinformatics. 2024 May 15;25:191. doi: 10.1186/s12859-024-05809-3 (PMC11095026; doi:10.1186/s12859-024-05809-3)
Supplement: Supplementary file 1 — Additional file1 (DOCX 21675 KB) [file 12859_2024_5809_MOESM1_ESM.docx]

Supplementary Material

Readsynth: short-read simulation for consideration of composition-biases in reduced metagenome sequencing approaches

Ryan Kuster*, Margaret Staton

*** Correspondence:** Ryan Kuster: rkuster@utk.edu

# Supplementary Figures and Tables

**Table S1.** Performance of simulations using various parameter settings using a metagenome of hypothetical gut microbial taxa. Wall clock time reported for all simulations.

| number of genomes | metagenome size (Gb) | number of reads (n) | motif1 (m1) | motif2 (m2) | cut rate (c) | max. length (bp) | error profile | time |
| --- | --- | --- | --- | --- | --- | --- | --- | --- |
| *692 | 5.846 | 3,321,470 | CATG/ | A/CGT | 1 | 1,006 | - | 204m36.979s |
| 691 | 2.582 | 3,196,330 | CATG/ | A/CGT | 1 | 1,006 | - | 111m59.763s |
| 691 | 2.582 | 49,541,809 | CATG/ | A/CGT | 1 | 1,006 | - | 113m37.994s |
| 691 | 2.582 | 50,339,998 | CATG/ | A/CGT | 0.8 | 600 | - | 65m5.266s |
| 691 | 2.582 | 49,819,185 | CATG/ | A/CGT | 1 | 600 | - | 54m34.212s |
| 691 | 2.582 | 49,819,185 | CATG/ | A/CGT | 1 | 600 | novaseq | 75m2.522s |
| 691 | 2.582 | 49,993,869 | G/AATTC | A/CCGGT | 1 | 1,006 | - | 16m22.367s |
| 691 | 2.582 | 49,996,665 | G/AATTC | A/CCGGT | 0.8 | 600 | - | 13m8.017s |
| 691 | 2.582 | 49,999,426 | G/AATTC | A/CCGGT | 1 | 600 | - | 11m50.082s |
| 691 | 2.582 | 49,999,426 | G/AATTC | A/CCGGT | 1 | 600 | miseq | 28m38.893s |

*metagenome includes host human genome reference

**Table S2.** Profiling results of reads simulated from HELIUS metagenomic community using various restriction enzymes, read count, and size selection. The reported Pearson correlation coefficients (r) for the taxonomic ratio, mean, and median estimates of relative abundance are reported when available.

| Simulation Settings | | | | | Percent Recovery | | | Pearson r vs. Ground Truth | | |
| --- | --- | --- | --- | --- | --- | --- | --- | --- | --- | --- |
| RE1 | **RE2** | **Mean** | **Reads** | **Fragments** | **Reads** | **Fragments** | **Taxa** | **Ratio** | **Mean** | **Median** |
| ecori | agei | 406 | 1.E+05 | 22,100 | 68.97% | 69.78% | 27.38% | 1.00 | 0.87 | 0.89 |
| ecori | msei | 406 | 1.E+05 | 35,552 | 64.12% | 70.48% | 15.74% | 1.00 | 0.98 | 0.91 |
| hhai | agei | 406 | 1.E+05 | 33,578 | 67.51% | 73.46% | 15.41% | 1.00 | 0.99 | 0.84 |
| hhai | msei | 406 | 1.E+05 | 39,373 | 61.09% | 71.30% | 6.23% | 0.99 | 0.99 | 0.98 |
| ecori | agei | 406 | 1.E+06 | 55,577 | 68.96% | 70.09% | 68.85% | 1.00 | 0.91 | 0.95 |
| ecori | msei | 406 | 1.E+06 | 113,506 | 64.69% | 70.72% | 46.72% | 1.00 | 0.96 | 0.85 |
| hhai | agei | 406 | 1.E+06 | 99,462 | 67.75% | 72.41% | 44.10% | 1.00 | 0.87 | 0.75 |
| hhai | msei | 406 | 1.E+06 | 199,827 | 64.25% | 72.75% | 26.72% | 1.00 | 0.90 | 0.74 |
| ecori | agei | 406 | 1.E+07 | 96,354 | 65.01% | 66.74% | 91.97% | 1.00 | 0.66 | 0.52 |
| ecori | msei | 306 | 1.E+07 | 342,408 | 64.44% | 68.27% | 89.02% | 1.00 | 0.99 | 0.90 |
| ecori | msei | 406 | 1.E+07 | 267,416 | 63.21% | 68.73% | 96.07% | 1.00 | 0.92 | 0.59 |
| ecori | msei | 506 | 1.E+07 | 204,873 | 62.38% | 69.98% | 96.72% | 1.00 | 0.89 | 0.46 |
| hhai | agei | 406 | 1.E+07 | 222,889 | 65.88% | 70.89% | 93.44% | 1.00 | 0.78 | 0.64 |
| hhai | msei | 406 | 1.E+07 | 548,629 | 64.27% | 70.68% | 66.72% | 1.00 | 0.92 | 0.48 |
| ecori | agei | 406 | 1.E+08 | 121,592 | 60.56% | 62.13% | 92.30% | 1.00 | 0.93 | 0.68 |
| ecori | msei | 406 | 1.E+08 | 402,706 | 61.90% | 65.47% | 97.54% | 1.00 | 0.95 | 0.39 |
| hhai | agei | 406 | 1.E+08 | 329,159 | 57.15% | 67.56% | 92.95% | 1.00 | 0.61 | 0.43 |
| hhai | msei | 406 | 1.E+08 | 1,132,631 | 60.30% | 68.03% | 97.54% | 1.00 | 0.98 | 0.68 |
| bcgi | bcgi | 34 | 1.E+07 | 532,599 | 47.63% | 54.35% | 56.07% | NA | 1.00 | 1.00 |

**A**


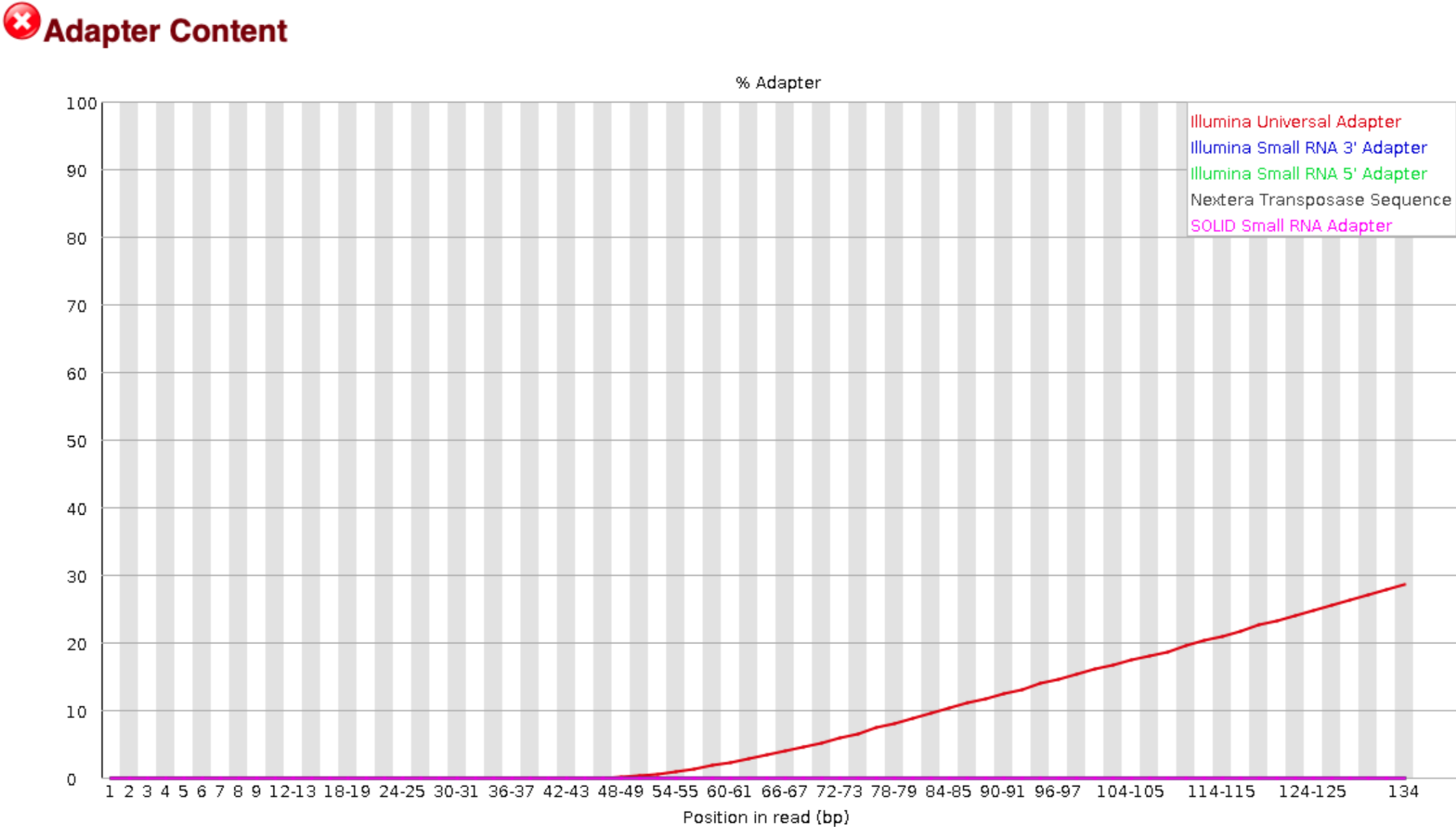


**B**


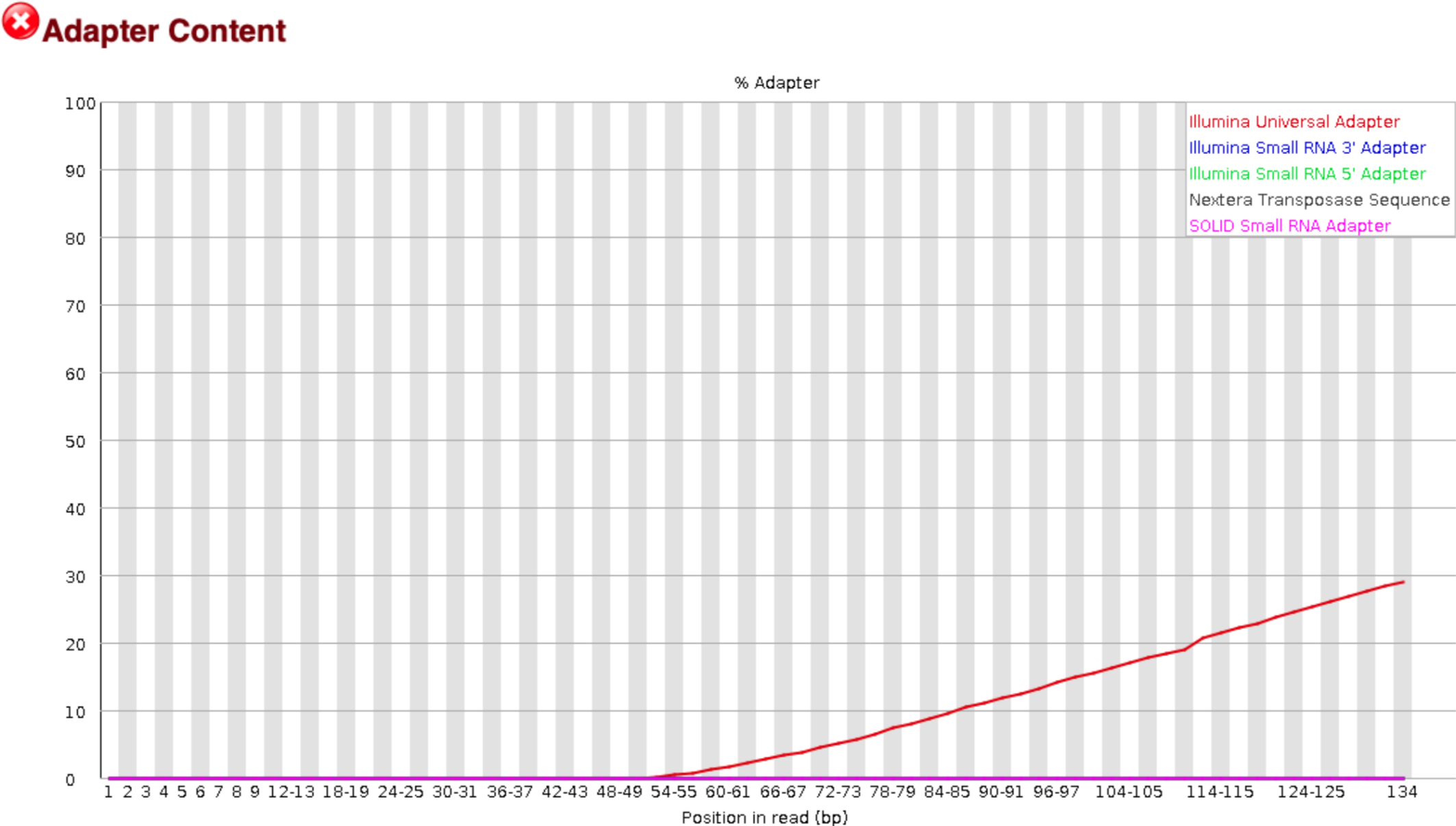


**Supplementary Figure 1.** Fastqc adapter content for simulated reduced metagenome sequencing (RMS) reads (A) and the real reads the simulations were derived from (B).

**A**


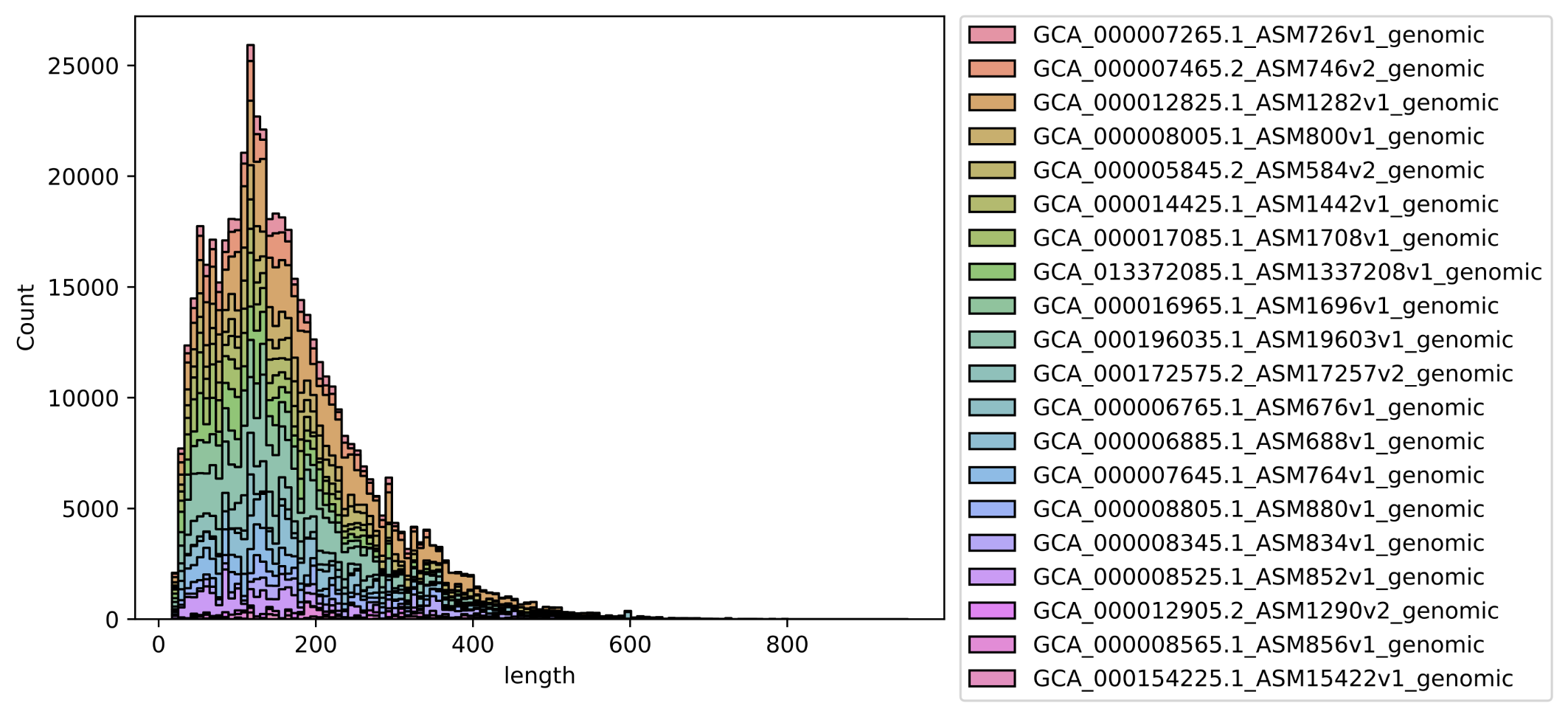


**B**


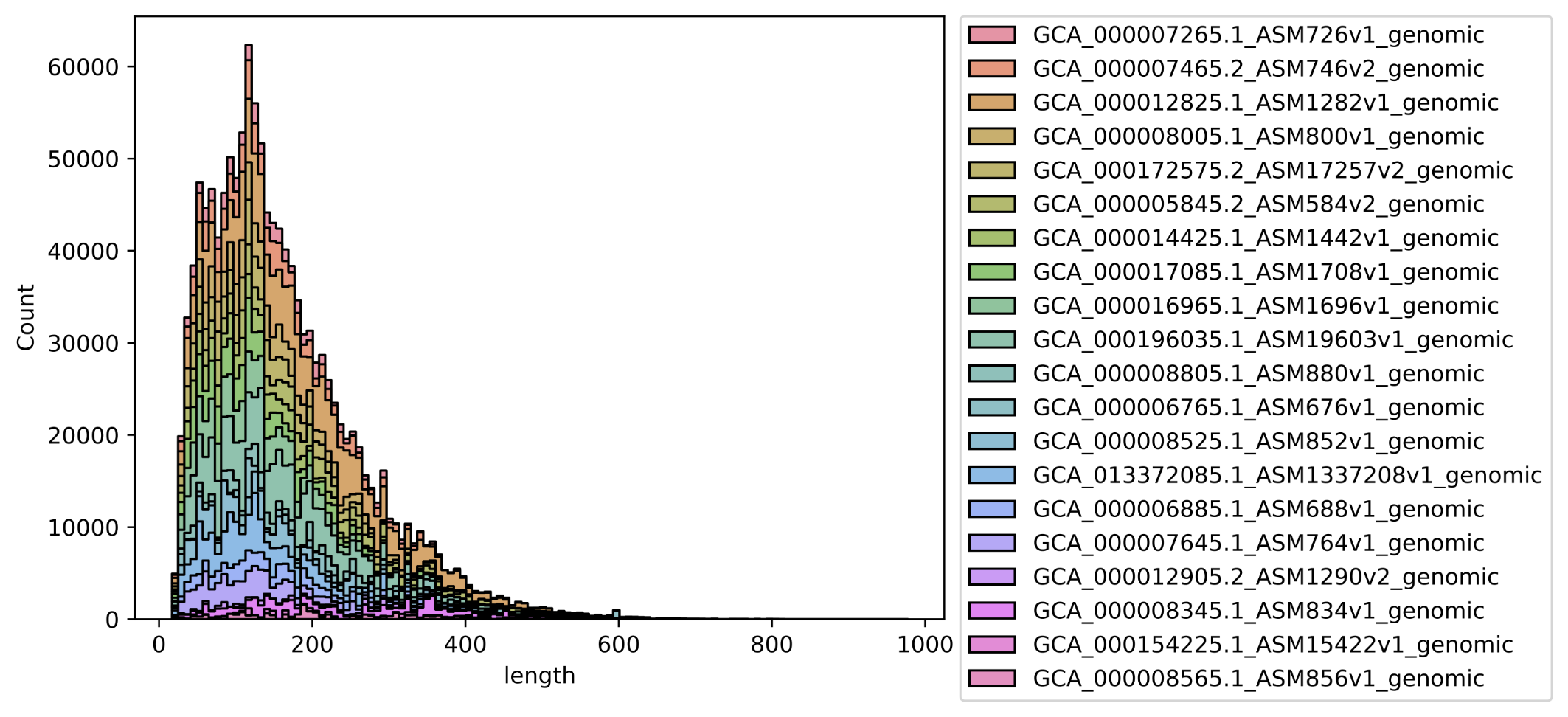


**C**


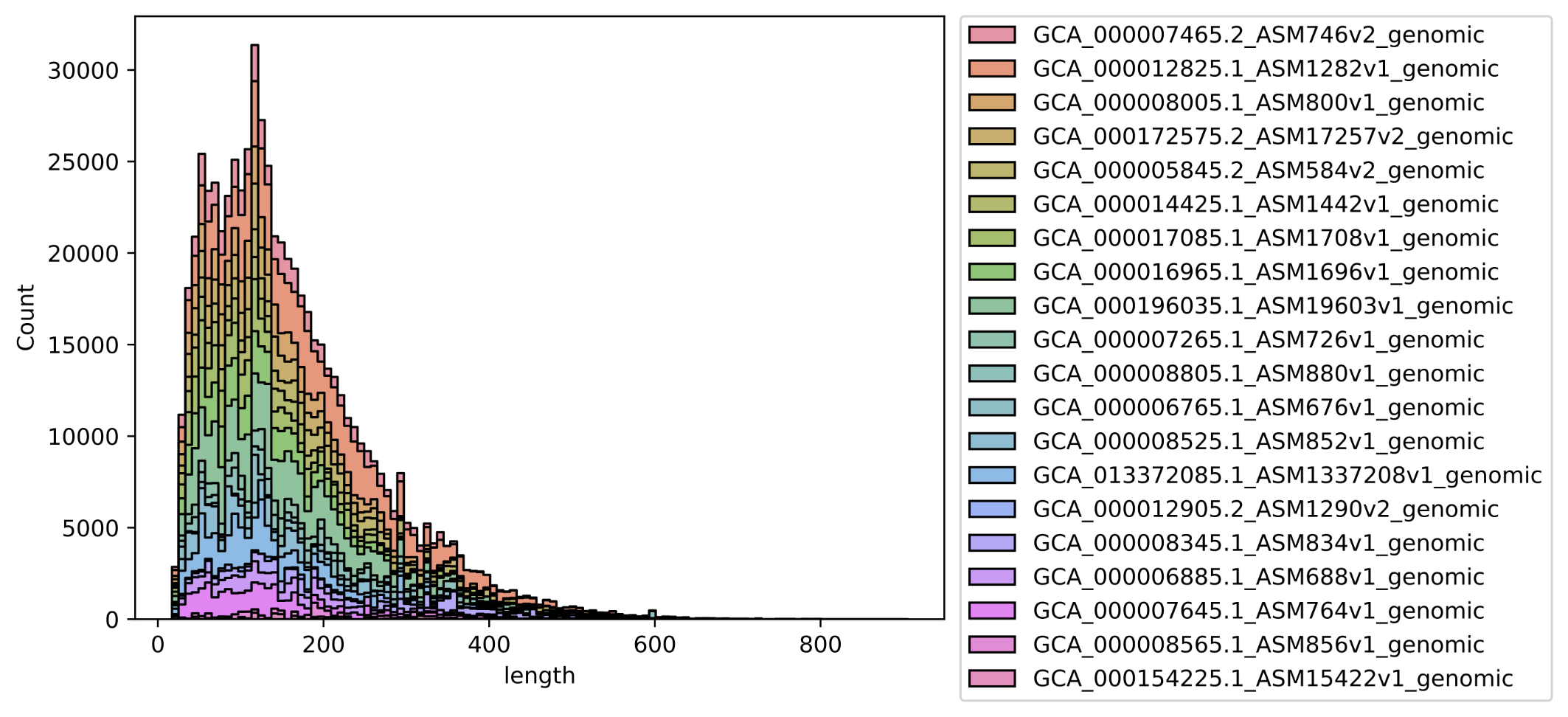


**Supplementary Figure 2.** Fragment distribution recreation from BWA MEM mapped reads from three replicates (A-C) of existing reduced metagenome sequencing (RMS) reads of a known mock community.


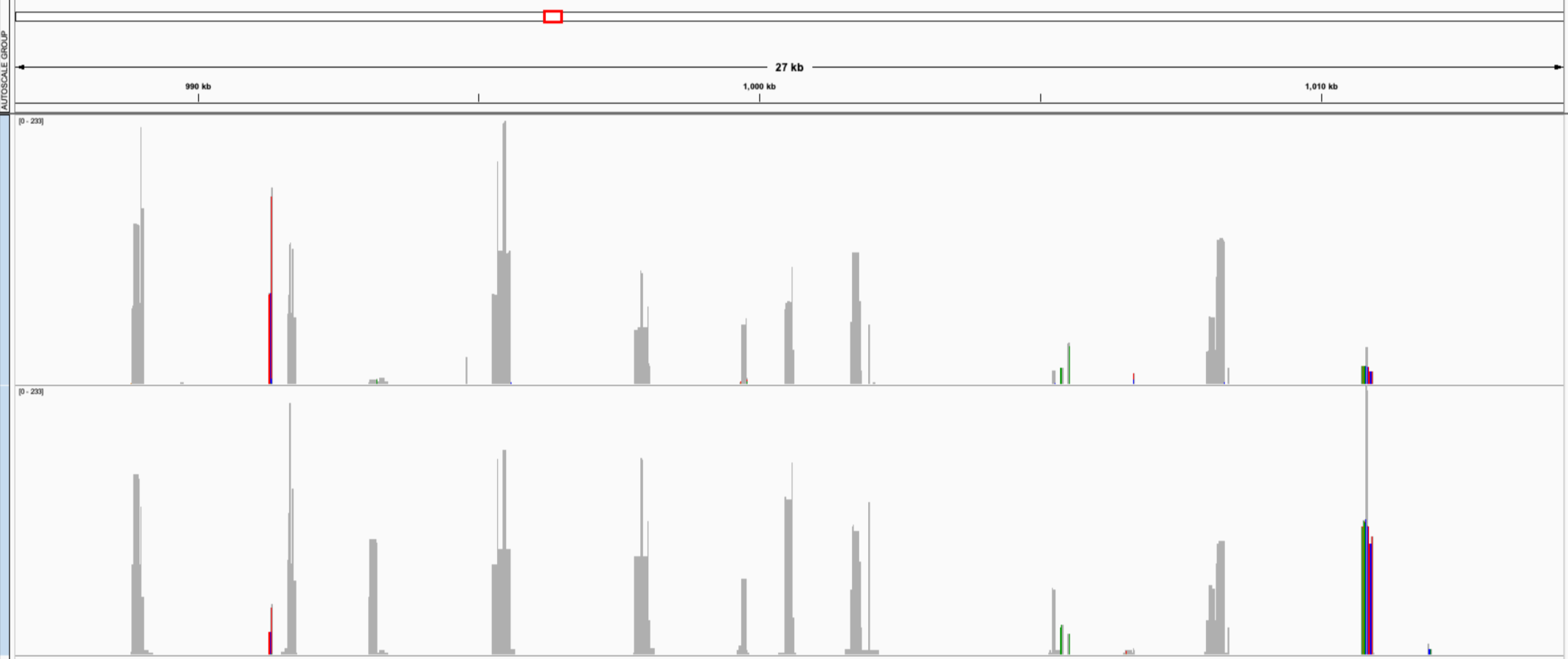


**Supplementary Figure 3.** Example IGV visualization of BWA MEM aligned read depth comparison (autoscaled). The top and bottom tracks are the real and simulated reads, respectively. Aligned reads with non-gray bars likely correspond with sequences aligned from similar taxa in the mock community or erroneous alignment.


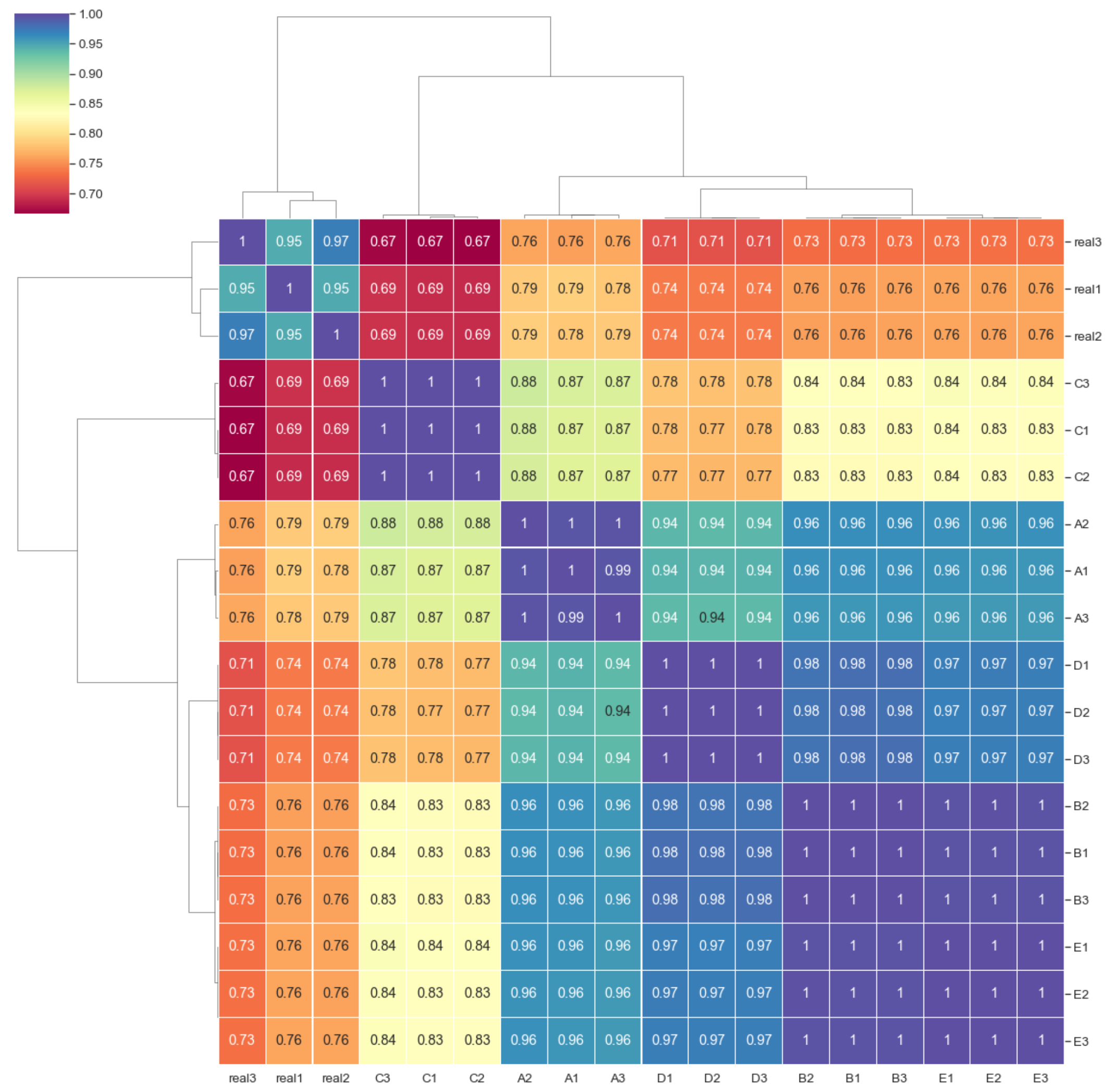


**Supplementary Figure 4.** Pearson correlation coefficient of metagenome-wide, position-specific read depth for each of three replicates of real and simulated mock community sequencing. Real1-3 are the depth correlations of the real sequencing data replicates. Mapped read depth correlations from readsynth simulations are shown in A-E: **A** - custom (.json) dictionary of fragment lengths derived from real sequence data and cut efficiency derived from real sequence data; **B** - normal distribution of fragment lengths and cut efficiency derived from real sequence data; **C** - normal distribution of fragment lengths with fragment mean increased by 100bp and cut efficiency derived from real sequence data; **D** - normal distribution of fragment lengths and cut efficiency reduced to 0.8; **E** - normal distribution of fragment lengths and cut efficiency increased to 1.

**A**


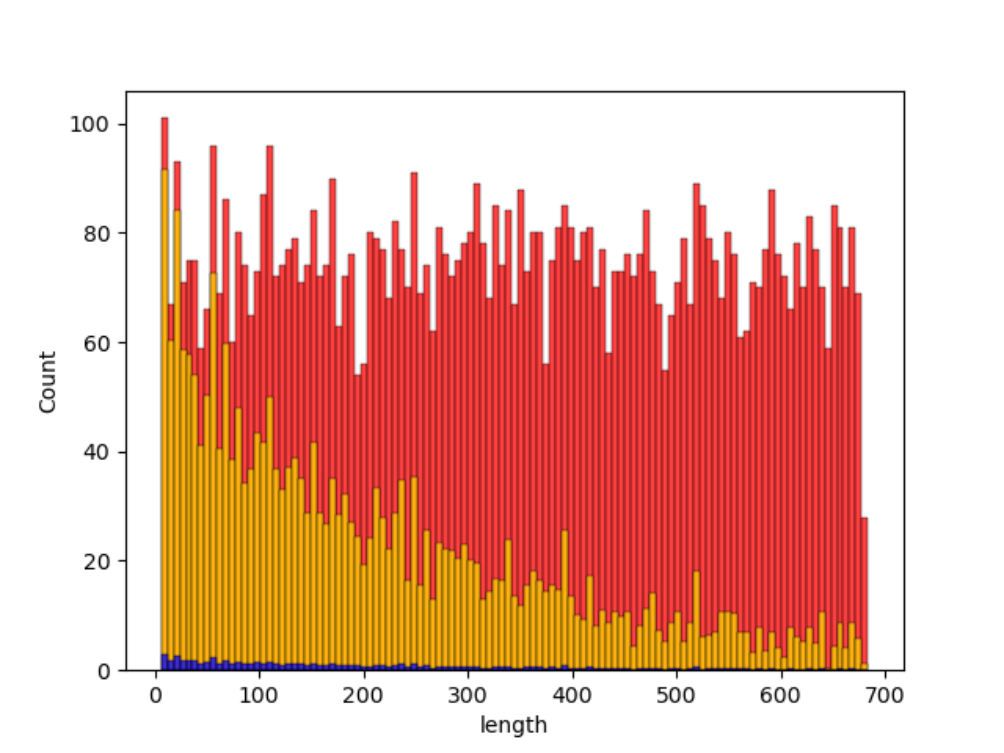


**B**


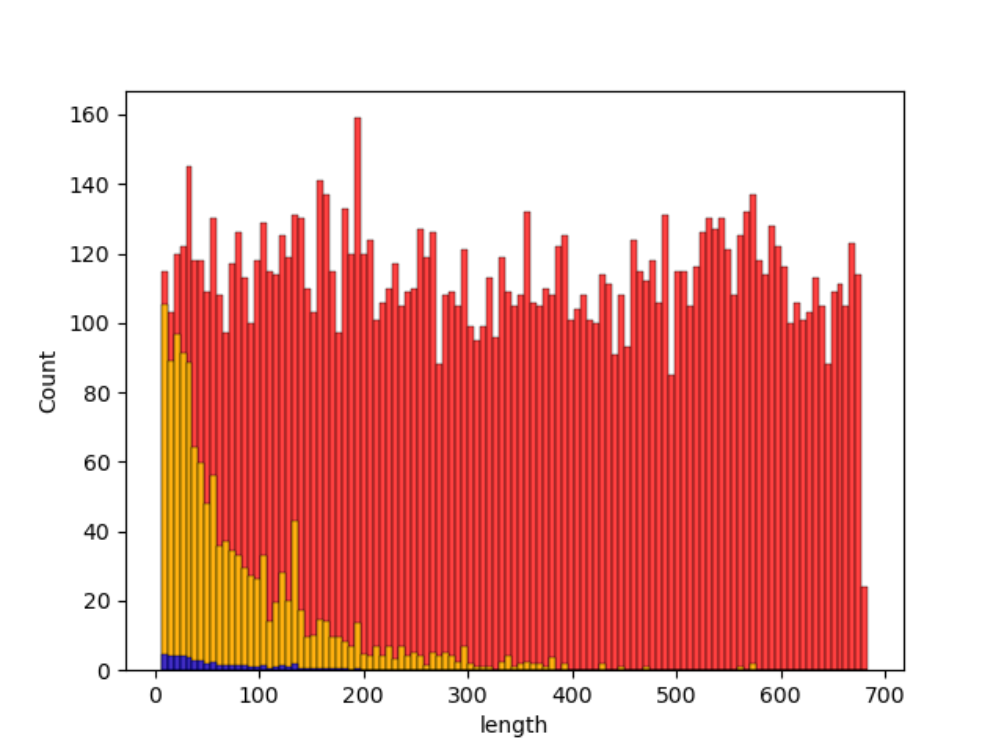


**Supplementary Figure 5.** Simulated expected fragment length distribution of two disproportionately represented taxa A: *Phocaeicola vulgatus*, and B: *Staphylococcus epidermidis* using identical library preparation and 1X genome copy number. Red bars represent the possible fragments in the size range while yellow fragments are the estimated frequency of fragments based on enzyme cut efficiency. These counts are then scaled based on the defined relative abundance (blue).


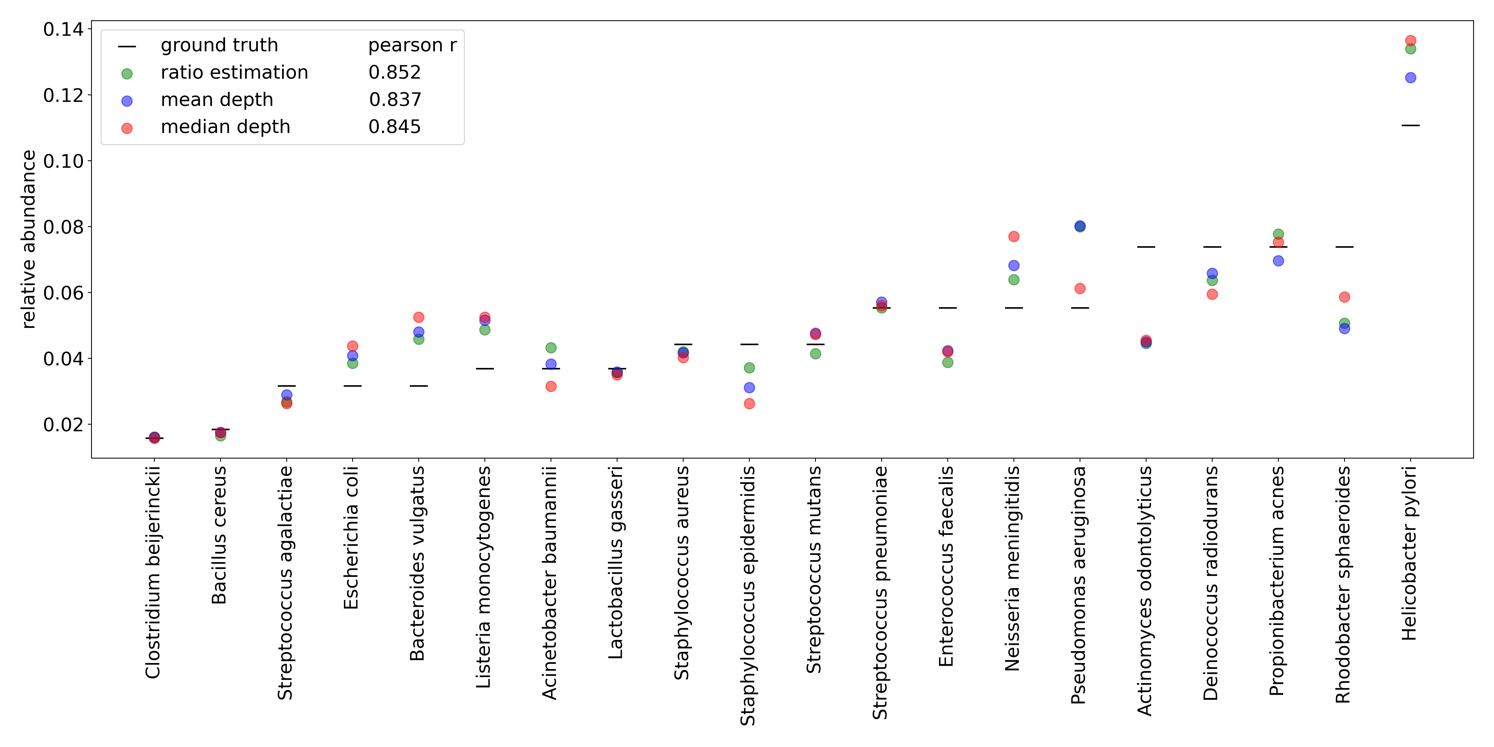


**Supplementary Figure 6.** Comparisons of ground truth relative abundance vs. results obtained using taxonomic ratio approach, mean depth, and median depths for SRR10199716 replicate of the Snipen et al. 2021 mock community sequences.


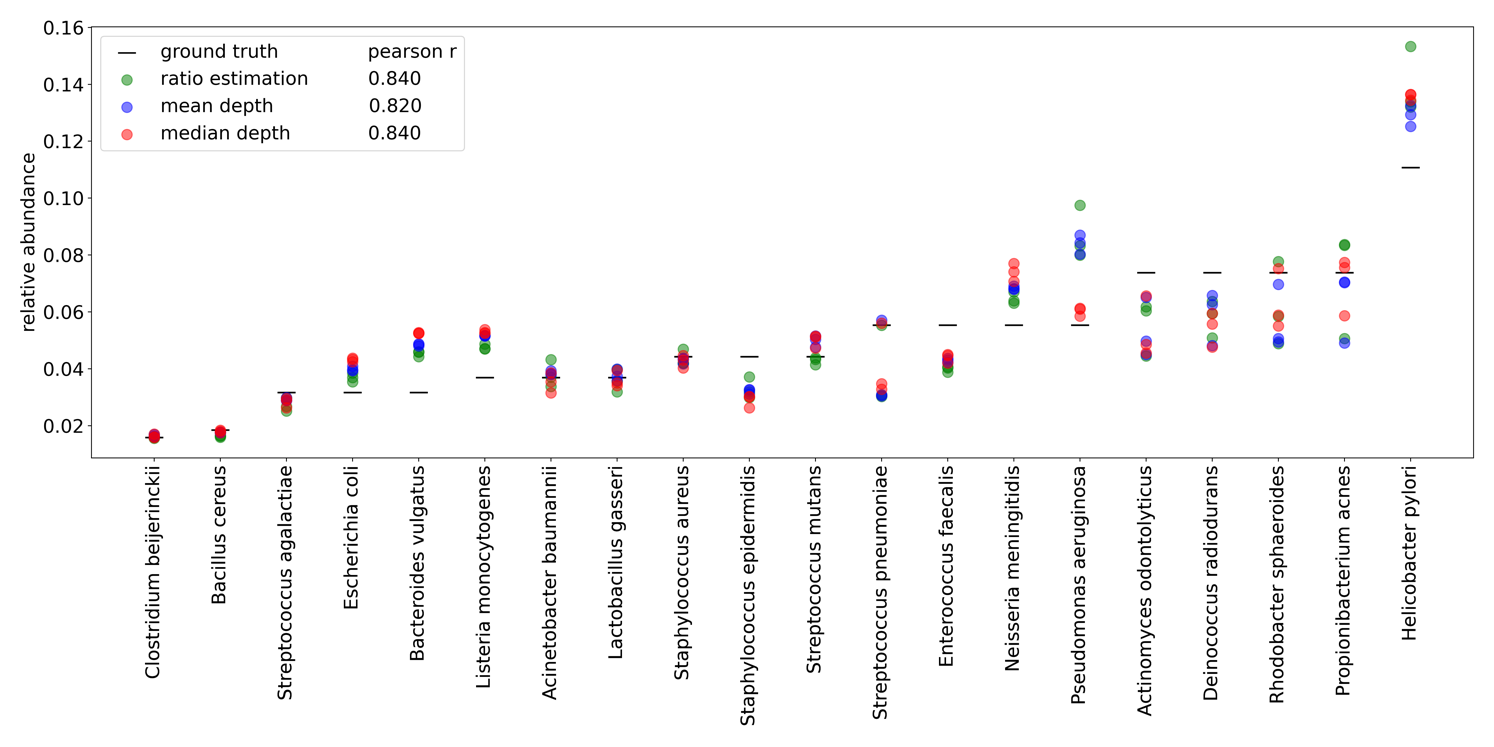


**Supplementary Figure 7.** Relative abundance comparisons between multiple prediction metrics and the ground truth over three replicates of Snipen et al. RMS mock community reads after fragment reconstruction. Estimates of relative abundance show consistent patterns of over-and underabundance in a taxa-dependent manner in some of the 20 mock community members using the ratio (green), mean (blue), and median (red) metrics for estimation.


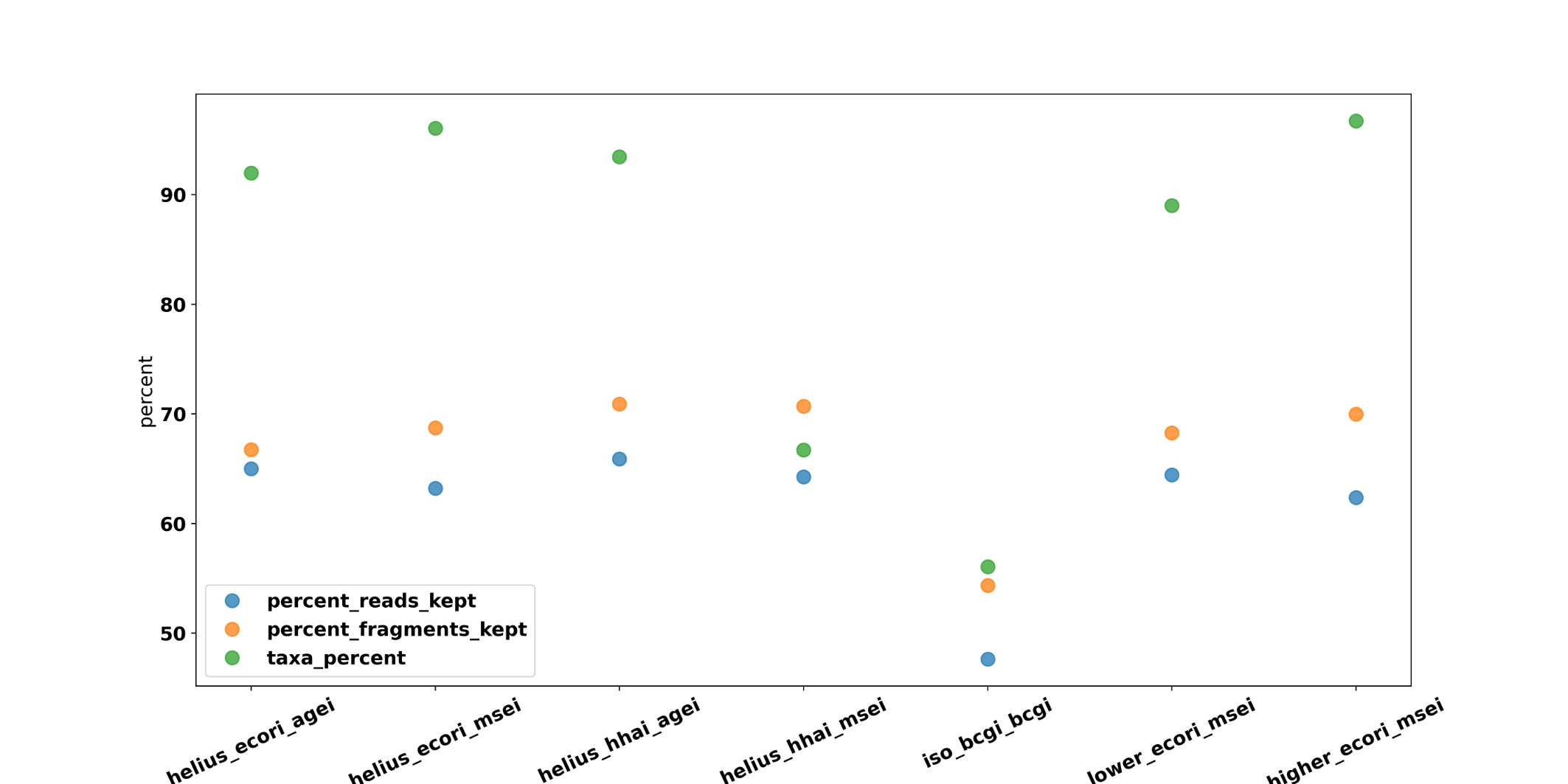


**Supplementary Figure 8.** Comparison of performance of multiple reduced representation metagenomic sequencing efforts simulated at 10^7^ target reads based on the same HELIUS community composition. The percent of identifiable taxa (“taxa_percent”) is lowest in the libraries simulated using either the combination of HhaI/MseI or the type IIb restriction enzyme BcgI. The percent of expected, unique fragments (“percent_fragments_kept”) that are retained after BWA mapping unique reads by these methods is comparable in all but the BcgI library (iso_bcgi_bcgi). Datasets were also simulated using mean fragment lengths lowered by 100bp (lower_ecori_msei) or increased by 100bp (higher_ecori_msei) relative to the baseline values used for the other double digestion simulations.


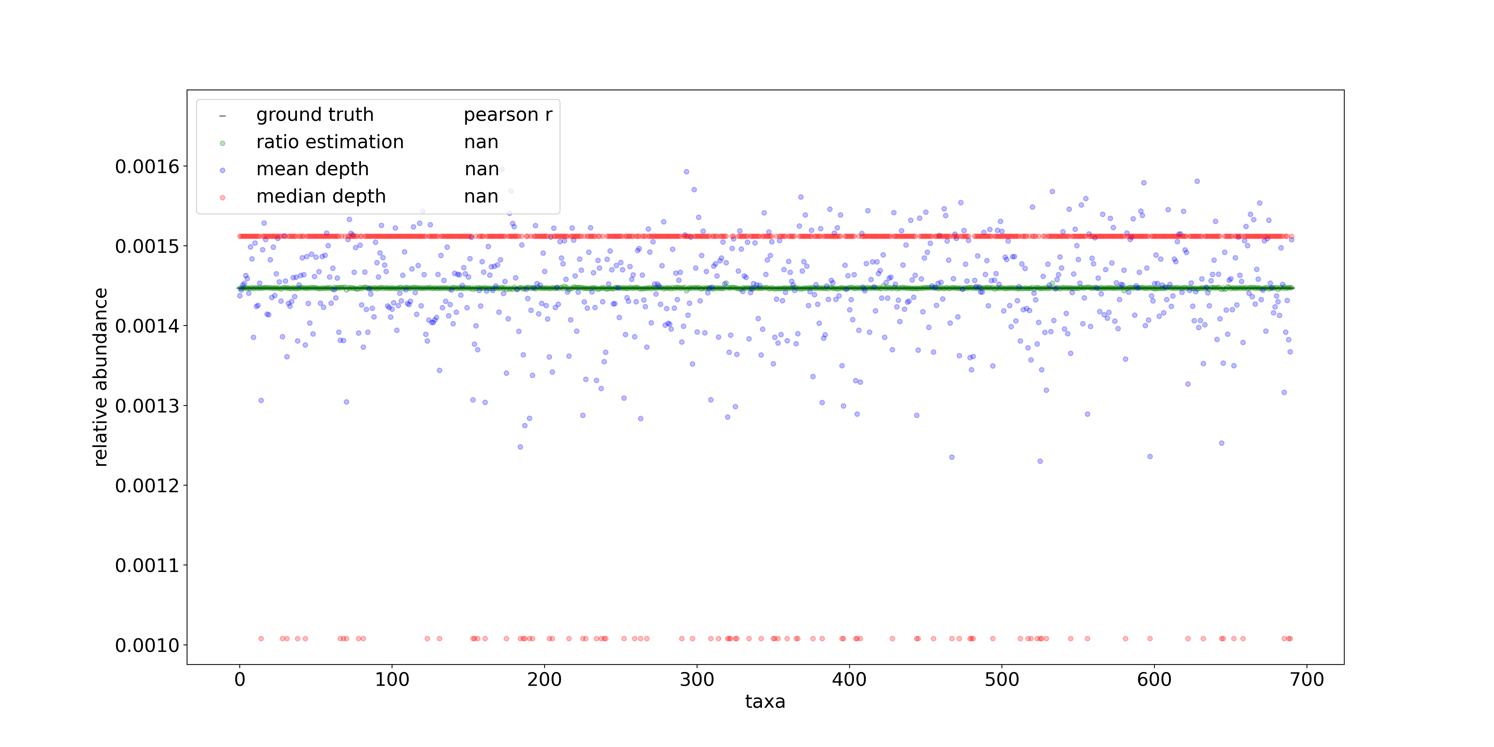


**Supplementary Figure 9.** Comparisons of ground truth vs. results obtained using taxonomic ratio approach, mean depth, and median depths by mapping simulated reads to the known set of community reference genomes before fragment recreation. Reads from 691 taxa were simulated based on the metagenomic taxa from Liu et al. 2017 sequence SRR5298272 using even representation. *In silico* reference genomes were digested with NlaIII and HpyCH4IV producing ~3.3x10^6^ total reads.


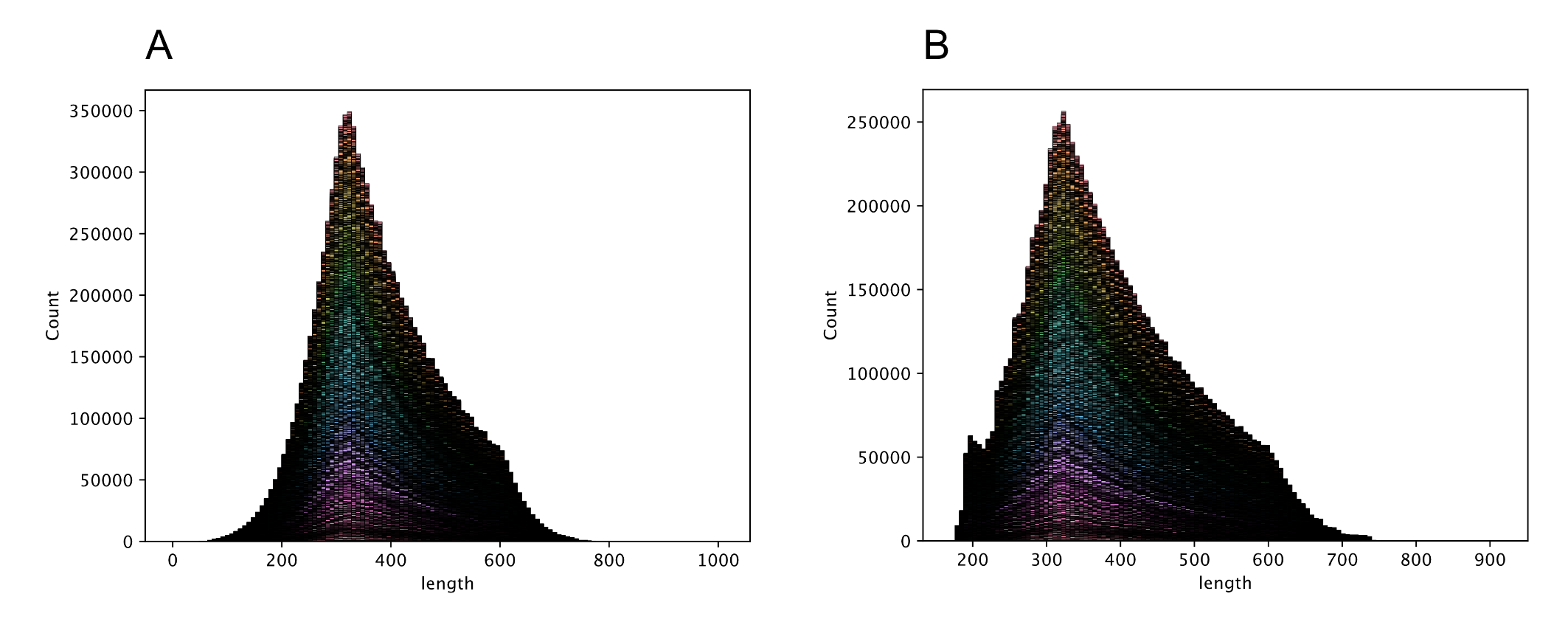


**Supplementary Figure 10.** Visual comparison of the fragment lengths from simulated Liu et al. data from the A) expected, known distribution and the B) recreated distribution mapped using a fully-inclusive ‘PlusPFP’ Kraken2 database (93.8% of simulated reads mapped).
